# Enhancement of Pathogen Resistance in Common Bean Plants by Inoculation With *Rhizobium etli*

**DOI:** 10.3389/fpls.2019.01317

**Published:** 2019-10-22

**Authors:** Armando Díaz-Valle, Alberto Cristian López-Calleja, Raúl Alvarez-Venegas

**Affiliations:** Centro de Investigación y de Estudios Avanzados del Instituto Politécnico Nacional, Unidad Irapuato, Guanajuato, Mexico

**Keywords:** induced systemic resistance, priming, nodule, *Rhizobium etli*, *Pseudomonas*

## Abstract

Symbiotic *Rhizobium*-legume associations are mediated by exchange of chemical signals that eventually result in the development of a nitrogen-fixing nodule. Such signal interactions are thought to be at the center of the plants’ capacity either to activate a defense response or to suppress the defense response to allow colonization by symbiotic bacteria. In addition, the colonization of plant roots by rhizobacteria activates an induced condition of improved defensive capacity in plants known as induced systemic resistance, based on “defense priming,” which protects unexposed plant tissues from biotic stress.Here, we demonstrate that inoculation of common bean plants with *Rhizobium etli* resulted in a robust resistance against *Pseudomonas syringae* pv. *phaseolicola*. Indeed, inoculation with *R. etli* was associated with a reduction in the lesion size caused by the pathogen and lower colony forming units compared to mock-inoculated plants. Activation of the induced resistance was associated with an accumulation of the reactive oxygen species superoxide anion (O_2_^−^) and a faster and stronger callose deposition. Transcription of defense related genes in plants treated with *R. etli* exhibit a pattern that is typical of the priming response. In addition, *R. etli*–primed plants developed a transgenerational defense memory and could produce offspring that were more resistant to halo blight disease. *R. etli* is a rhizobacteria that could reduce the proliferation of the virulent strain *P. syringae* pv. *phaseolicola* in common bean plants and should be considered as a potentially beneficial and eco-friendly tool in plant disease management.

## Introduction

Over the last few decades, particularly after the Green Revolution, the global agricultural production increased considerably. However, due to the sustained increase in the global human population, the demand for higher crop production has also increased substantially. Accordingly, to achieve higher agriculture yields, farmers have adopted the extensive application of chemical fertilizers and pesticides, resulting in soil degradation and decrease in soil fertility (Gouda et al., 2018). In addition, nitrous oxide (N_2_O), which is a by-product of excess nitrogen (N) fertilization, is a chemical pollutant and contributes to global warming as it is the single most important ozone-depleting substance (Ravishankara et al., 2009; Vejan et al., 2016). Also, the excess application of ammonium nitrate in soils leads to a decline in symbiotic interactions established between microbes and legume plants (Vejan et al., 2016). Therefore, reducing the use of synthetic chemical pesticides and fertilizers is one of the challenges in modern society.

Some of the strategies involved in sustainable crop production and integrated pest management include development of novel cultivars exhibiting disease resistance, abiotic stress tolerance, and high nutritional value, application of genetic engineering tools to develop non-legume crops participating in N-fixing symbioses, and the use of biological control agents and plant growth promoting rhizobacteria (PGPR) (Bruce, 2010; Ubertino et al., 2016; Gouda et al., 2018). The use of PGPR is one of the most promising tools for disease management in contemporary agriculture and an alternative that could minimize reliance on agrochemicals (Timmermann et al., 2017). PGPR can be used to promote plant growth and development and enhance plant health without negatively affecting natural ecosystems and biodiversity and without environmental contamination (Calvo et al., 2014; Vejan et al., 2016). With regard to their association with root cells, PGPR could be classified into either intracellular PGPR (iPGPR, symbiotics) or extracellular PGPR (ePGPR, free living) (Martínez-Viveros et al., 2010). iPGPR may inhabit specialized root structures such as root nodules, while ePGPR may be found on root surfaces, in the rhizosphere, or in between root cortex cells (Martínez-Viveros et al., 2010; Gouda et al., 2018).

PGPR have numerous direct or indirect mechanisms for preventing growth and development of pathogens, such as antagonism, competitive exclusion, production of antibiotics, or extracellular secondary metabolites that inhibit pathogen growth, signal interference, competition for nutrients/resources (e.g., fixed N, ferric ion by siderophore production), hormone production, and the activation of the induced systemic resistance (ISR) plant defense system (Haas and Défago, 2005; Glick, 2012; Timmermann et al., 2017).

ISR is defined as an induced condition of enhanced defensive capacity in plants, which is activated in response to biological or chemical stimuli (e.g., colonization of the roots by PGPR or beneficial fungi or treatment with chemical compounds), and protects unexposed plant tissues against pathogenic invasion and insect herbivory (Pieterse et al., 2014; Timmermann et al., 2017; Gouda et al., 2018). ISR aids plants to fight numerous diseases and is effective against a wide range of pathogenic bacteria and fungi, and the activation of the defense mechanisms acts systemically in plant sections that are spatially separated from the inducer (Pieterse et al., 2014).

Activation of ISR by advantageous microorganisms is often based on priming (Pieterse et al., 2014), a physiological state that prepares the plant for a more rapid and/or greater activation of cellular defenses when exposed to biotic or abiotic stress, resulting in an enhanced level of resistance (Conrath, 2009; Conrath, 2011). In 1991, Van Peer and colleagues revealed that priming of defense responses is involved in ISR. In their study, the roots of carnation plants (*Dianthus caryophyllus*) were “bacterized” (primed) with *Pseudomonas* sp. strain WCS417r prior to inoculation with the pathogen *Fusarium oxysporum* f. sp. *dianthi*. Primed plants (bacterized) showing ISR exhibited enhanced synthesis and accumulation of phytoalexins following inoculation with *F. oxysporum*, when compared with non-induced control plants (Van Peer et al., 1991). Furthermore, induced resistance against pathogens has been associated with a faster and stronger accumulation of callose (Ton et al., 2005). For example, β-amino-butyric-acid (BABA) induced resistance in *Arabidopsis* correlates with primed deposition of callose-rich papillae (or enhanced accumulation of callose; Ton and Mauch-Mani, 2004).

Callose-containing cell-wall appositions, named papillae (where antimicrobial compounds can be deposited; Luna et al., 2011), are induce at the sites of pathogen attack, in primed plants, at early stages of pathogen invasion, and could also contribute to disease resistance by strengthening the plant cell wall at the site of pathogen invasion (Kohler et al., 2002).

In *Arabidopsis* plants primed by ISR (following colonization by PGPR), activation of the induced response involves the activation of the salycilic acid (SA)–, jasmonate (JA)-, and ethylene (ET)-signaling pathways. For example, priming with the PGPR *Paraburkholderia phytofirmans* PsJN reduces plant susceptibility to disease and the proliferation of the virulent strain *Pseudomonas syringae* pv. tomato DC3000 (PstDC3000) (Timmermann et al., 2017). In addition, *P. phytofirmans* PsJN induces transcriptional changes in key defense-related genes of the SA-, JA-, and ET-signaling pathways (e.g., *PATHOGENESIS RELATED 1*, *PLANT DEFENSIN 1.2*) in plants infected with PstDC3000. Also, the plant hormone abscisic acid (ABA), which regulates numerous plant developmental processes and adaptive responses to stress, has been involved in priming by beneficial microbes for enhanced callose deposition (Ton et al., 2005; Asselbergh et al., 2008; Pieterse et al., 2014). Furthermore, brassinosteroids modulate plant defense responses against pathogens and protect plants from environmental stresses, independently of SA-mediated defense signaling and PR gene expression (Krishna, 2003; Bari and Jones, 2009).

However, the activation of the different hormone-signaling pathway(s) following the application of the ISR-inducing PGPR seems to be specific to the plant species, as well as the pathogen and PGPR involved (De Vleesschauwer and Höfte, 2009; Timmermann et al., 2017). Basic research on the application of PGPR and in the exploitation of existing tools and techniques for safer and productive agricultural practices remains limited (Gouda et al., 2018).

Legume plants, particularly the common bean (*Phaseolus vulgaris* L.), are great model systems for studying ISR, as well as defense-priming and plant-pathogen interactions. For instance, common bean interacts with symbiotic diazotrophic bacteria (rhizobia). The symbiotic interaction results in the formation of new plant organs, N-fixing nodules, which fix atmospheric N (Taté et al., 1994). Therefore, symbiotic N-fixing rhizobacteria have positive effects on the host plants, not only facilitating nutrient availability but also priming defense against biotic and abiotic stresses *via* activation of ISR (Franche et al., 2009; Gouda et al., 2018).

To determine if rhizobacteria elicits the ISR in common bean against *P. syringae* pv. *phaseolicola* (PspNPS3121), the causal agent of the halo blight disease, we evaluated the effect of using *R. etli* to induce resistance based on pathogen accumulation, symptom emergence, nodule number, N fixation, callose deposition, and changes in levels of expression of defense related genes, within and across generations.

## Materials and Methods

### Plant Material

*P. vulgaris* cultivar “Rosa de Castilla” (Jiménez-Hernández et al., 2012), a type of bean produced under rainfed conditions and susceptible to the halo blight bacterial disease, was used in the present study. Seeds were surface sterilized (15 min in 2% NaOCl), rinsed three times with sterile deionized water, and germinated under sterile conditions, in moist paper towels, at 28°C. Three days after germination (dag), the seedlings were transferred to 2.3-L pots containing vermiculite, inoculated with 4 ml of the *Rhizobium etli* CE3 suspension, and placed in a greenhouse (Guanajuato, México, 101°09′01″ W, 20°30′09″ N; 1730 masl; as described by Martínez-Aguilar et al., 2016). The experiments were performed under daylight conditions, with maximum and minimum temperatures of 30 and 18°C, respectively. All inoculated plants were fertilized once a week with a B&D nutrient solution (Broughton and Dilworth, 1971) without N. Non-inoculated plants were fertilized with a B&D solution supplemented with N (8 mM KNO_3_; Estrada-Navarrete et al., 2007).

### Rhizobacteria Growth Conditions and Inoculation

*R. etli* strain CE3 was grown at 28°C on PY media (5 g/L of peptone, 3 g/L of yeast extract, 0.7 g/L of calcium chloride) supplemented with 20 μg/ml nalidixic acid, 100 mg/ml streptomycin (Cárdenas et al., 2006), and 7 mM CaCl_2_ until the bacterial culture reached an OD_600_ of 0.5–0.6. The bacterial culture was centrifuged (5,000 rpm; Sorvall GSA Rotor), the pellets washed in 10 mM MgSO_4_, and the cells resuspended in 10 mM MgSO_4_ (Cárdenas et al., 2006). Subsequently, 3-day-old roots from “Rosa de Castilla” cultivar were inoculated, by drench, with 4 ml of the bacterial resuspension.

### Pathogen Infection

Pathogen infection in F0 plants was performed as described by Martínez-Aguilar et al., 2016. Briefly, *P. syringae* pv. *phaseolicola* NPS3121 (PspNPS3121) was cultivated for 36 h at 28°C on KB media containing 50 μg/ml rifampicin; then, the cells were resuspended in MgCl_2_ (10 mM) at a final concentration of 5 × 10^8^ colony-forming units per milliliter (CFU/ml). Four milliliters of the bacterial solution were placed in a syringe without a needle, and the abaxial surfaces of the second trifoliate leaves were infiltrated with the pathogen 17 dag. Six infiltration points per leaf were employed. Negative control plants were neither treated nor infiltrated, while positive control plants were not infiltrated but treated with *R. etli*. To determine the systemic effect, samples were obtained from distal leaves not exposed to the pathogen, 1 day before infection, and 1 and 5 days after infection. **Table 1** presents an outline of the experimental design.

**Table 1:** Experimental design.

Treatments	
*Rhizobium etli* (3 dag)	+ *Phaseolus syringae* (17 dag)
*R. etli* (3 dag)	+ H_2_O (17 dag)
*R. etli* (3 dag)	—
—	*P. syringae* (17 dag)
—	—

dag, days after germination.

### Disease Evaluation

Bacterial growth was assessed 10 days after infection as follows: three leaf discs adjacent to the infection sites were excised using a 1-cm diameter stainless steel cork borer, then rinsed and homogenized with sterile deionized water, and plated in serial dilutions on KB media containing 50 μg/ml rifampicin and 6 mM MgSO_4_. The plates were incubated for 36 h at 28°C, and the total numbers of CFUs from three plates for each dilution were counted. The percentage of leaf damage (total chlorotic leaf area/total leaf area) and lesion size (total chlorotic and necrotic leaf area/total leaf area), on leaves that had been exposed to the pathogen, was determined using Fiji software (Schindelin et al., 2012).

### Callose Quantification

To determine callose deposition in leaves of common bean plants, we followed the protocol described by Luna et al. (2011) with slight modifications. Seventeen days old *P. vulgaris* plants (co-cultivated with the symbiont) were immersed, for 30 s, in a bacterial solution (MgCl_2_ 10 mM, Silwet L-77 0.05%, and PspNPS3121 at a concentration of 5 × 10^8^ CFU/ml). After 24 h of pathogen infection, the plants were destained for 24 h in 95% ethanol, washed in 0.07 M phosphate buffer (pH 9), and incubated for 24 h in 0.07 M phosphate buffer containing 0.01% aniline blue (Sigma–Aldrich, catalog #415049). Imaging of callose deposition in three plants per treatment, from two leaves per plant (and four discs per leaf, with a diameter of 1 cm each), was performed with an Olympus BX50 microscope (UV filter BP 340–380 nm, LP 425 nm), and pictures were taken with a Lumenera INFINITY 3 camera. Quantification and callose intensity were determined by using the Fiji software (https://imagej.net) and following the protocol described by Jin and Mackey (2017).

### Histochemical Detection of Superoxide Radical

To detect the accumulation of the superoxide anion (O_2_^−^), a reactive oxygen species, we followed the protocol described by Timmermann et al. (2017) with some modifications. Aerial parts of 17 days old *P. vulgaris* plants (co-cultivated with the symbiont) were immersed, for 30 s, in a bacterial solution (MgCl_2_ 10 mM, silwett L-77 0.05%, and PspNPS3121 at a concentration of 5 × 10^8^ CFU/ml). After pathogen infection (24 h), leaves were removed from plants at 1.5, 3, 6, and 9 h and incubated for 24 h at room temperature in a phosphate buffered saline solution (137 mM NaCl; 2.7 mM KCl; 10 mM Na_2_HPO_4_; 2 mM KH_2_PO_4_) containing 0.06 mM nitroblue tetrazolium (Sigma–Aldrich, catalog # N6639). The leaves were then cleared in 80% ethanol (50 ml) at 60°C to extract chlorophylls (Timmermann et al., 2017). Imaging of purple formazan deposits (visualized as a blue precipitate), which result from the reaction of nitroblue tetrazolium with O_2_^−^ (Carvalho et al., 2008), identified the areas where O_2_^−^ was accumulated. Images were taken using a digital camera Nikon 5500 (Nikon Corp., Tokyo, Japan). Quantification of O_2_ was performed using the protocol described by Juszczak and Baier, 2014. Each experiment was independently performed in triplicate.

### Acetylene Reduction Assay

Acetylene reduction assays were performed using whole nodulated roots 24 days post-inoculation (dpi) according to Barraza et al. (2018). Each experiment was performed in triplicate. Nitrogenase-specific activity was expressed as nmol of C_2_H_2_ g^−1^ h^−1^ of nodule fresh weight (Vessey, 1994).

### Candidate Defense Genes With Enhanced Transcription in Response to Colonization of the Roots by *R. etli*

To identify genes in common bean that were potentially activated by ISR and that confer resistance to plant pathogens, we carried out searches in studies published on the subject of ISR and defense priming for the genes (according to Mayo et al., 2016). The following *P. vulgaris* genes were selected (orthologues of *Arabidopsis* genes): *ERF6* (*Phvul.002G055800*), *PR10* (*Phvul.002G209500*), *MYC2* (*Phvul.003G285700*), *NPR1* (*Phvul.006G131400*), *PR1* (*Phvul.006G196900*), *WRKY29* (*Phvul.002G293200*), *WRKY70* (*Phvul.009G043100*), *PAL2* (*Phvul.007G150500*), and *WRKY33* (*Phvul.008G251300*).

In addition, we analyzed the transcript levels of some of the genes coding for enzymes directly involved in plant hormone synthesis. Specifically, we determined the transcript levels of the gene orthologs to the *Arabidopsis* *AMI1* (*Phvul.007G180900*) and *NIT1* (*Phvul*.*011G096700*), both involved in auxin biosynthesis (Barraza et al., 2015b); *AAO3* (*Phvul.008G210000*), an aldehyde oxidase that catalyzes the last step of ABA biosynthesis and a marker for the sites of ABA production (Koiwai et al., 2004) and ABA biosynthesis in leaves (Seo et al., 2004); and *BR6OX2.2* (*Phvul.004G041700*), which catalyzes the last reaction in brassinosteroid (BR) biosynthesis (Kim et al., 2005). The importance of the genes was evaluated by the quantification of their transcripts *via* qPCR in response to *R. etli* and pathogen infection.

### Quantitative PCR (qPCR) Analysis

qPCR was performed according to Martínez-Aguilar et al. (2016). Data from qRT-PCR experiments were analyzed based on the relative quantification method, or the 2^−ΔΔCT^ method (Livak and Schmittgen, 2001). Fold changes in the target genes were normalized to *PvAct11* (Borges et al., 2012) and *PvTUB* (Livak and Schmittgen, 2001; Barraza et al., 2013, Barraza et al., 2015a), as endogenous controls, and relativized to the expression in control samples (relative gene expression in control plants was defined as 1). All experiments were run in triplicate (technical replicates) from three biological replicates. For a list of all primers used, see **Supplementary Table ST1**. Statistical significance was determined as described by Martínez-Aguilar et al. (2016).

### Transgenerational Inheritance of Priming

Additional experiments were conducted in the F1 progeny to explore the transgenerational priming effect. First, all common bean plants (F0 generation) from all the different treatments (rhizobacteria only, rhizobacteria plus pathogen, pathogen only, rhizobacteria plus water, and without treatment) were self-pollinated and grown to seed set to generate the F1 progeny. Subsequently, for the transgenerational priming analysis, seeds from the F1 progenies were germinated and exposed to the pathogen only, without rhizobacteria treatment. Plants were inoculated with the pathogen at the age at which the parental lines were infected (17 dag). Samples from inoculated plants were obtained from distal leaves that had not been exposed to the pathogen 24 h before infection, and 24 h and 120 h after infection (or 16, 18, and 22 dag, respectively). All samples were obtained in triplicate and stored at −80°C.

## Results

### Inoculation With *R. etli Protects P. vulgaris* Against PspNPS3121

**Figure 1:**
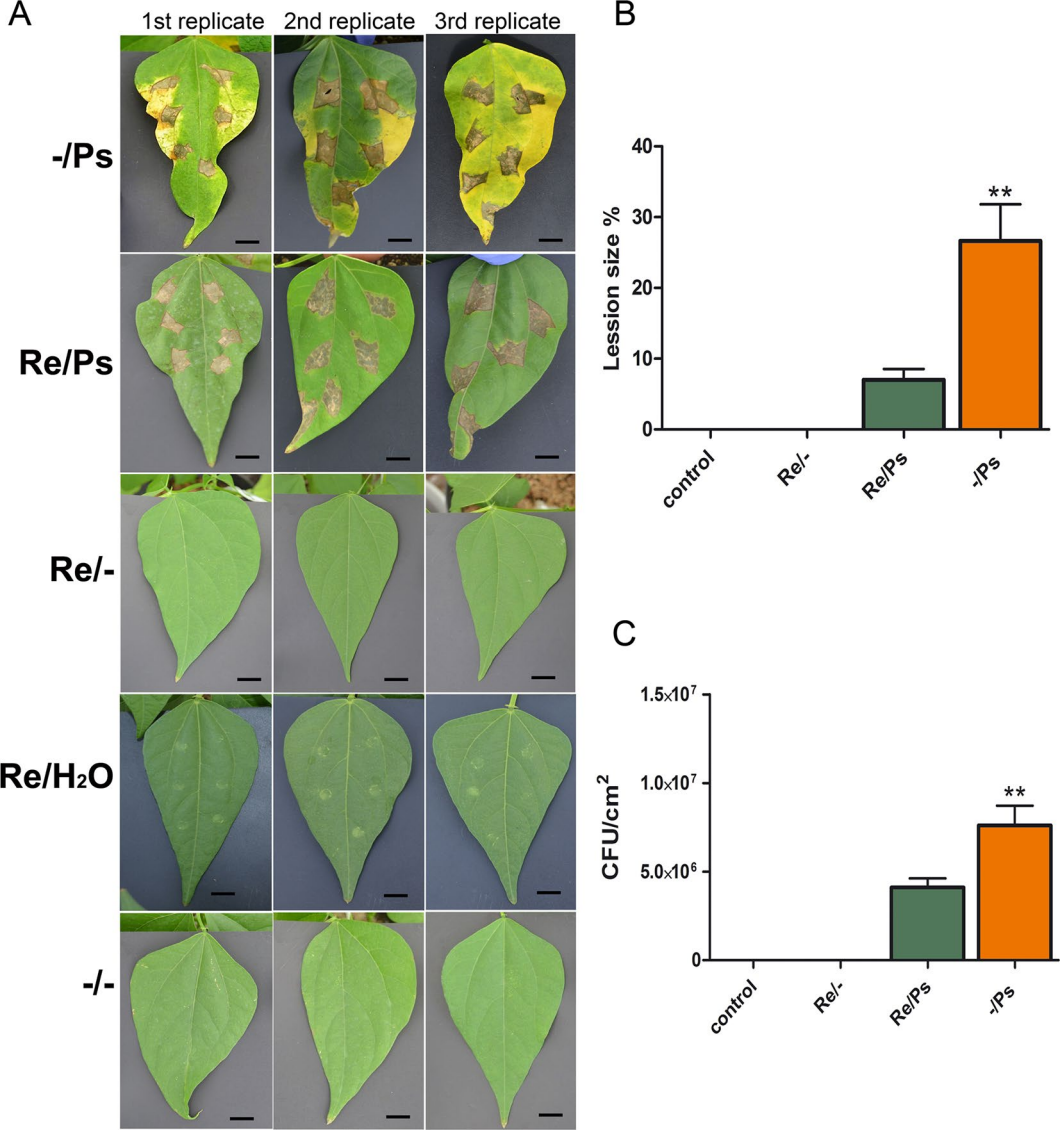
**(A)** Leaf damage in common bean plants inoculated with PspNPS3121 (Ps) after treatment with *R. etli* (Re), or water. Photos were taken 10 days after pathogen inoculation. **(B)** Lesion size (n = 26) and **(C)** colony forming units (CFU) of *Phaseolus vulgaris* plants 10 days after infection with PspNPS3121. Data are mean ± SEM from three independent experiments, and the statistical significance was evaluated using an unpaired two-tailed Student’s *t*-test (^**^ *p*< 0.05).

As shown in **Figure 1A**, inoculation of the symbiont *R. etli* in cultivar “Rosa de Castilla” before pathogenic bacteria inoculation resulted in a considerable increase in resistance to halo blight disease caused by PspNPS3121. Treatment with *R. etli* induced a 75% reduction in lesion size (Re/Ps), compared to plants treated with the pathogen only (-/Ps; **Figure 1B**). Also, bacterial populations in leaves from plants treated with *R. etli* and exposed to the pathogen (Re/Ps) were significantly lower, approximately 50% less, than in plants treated with the pathogen only (-/Ps; **Figure 1C**). We inferred that common bean plants treated with the *R. etli* were efficiently protected against PspNPS3121, in contrast to plants that were not primed.

**Figure 2:**
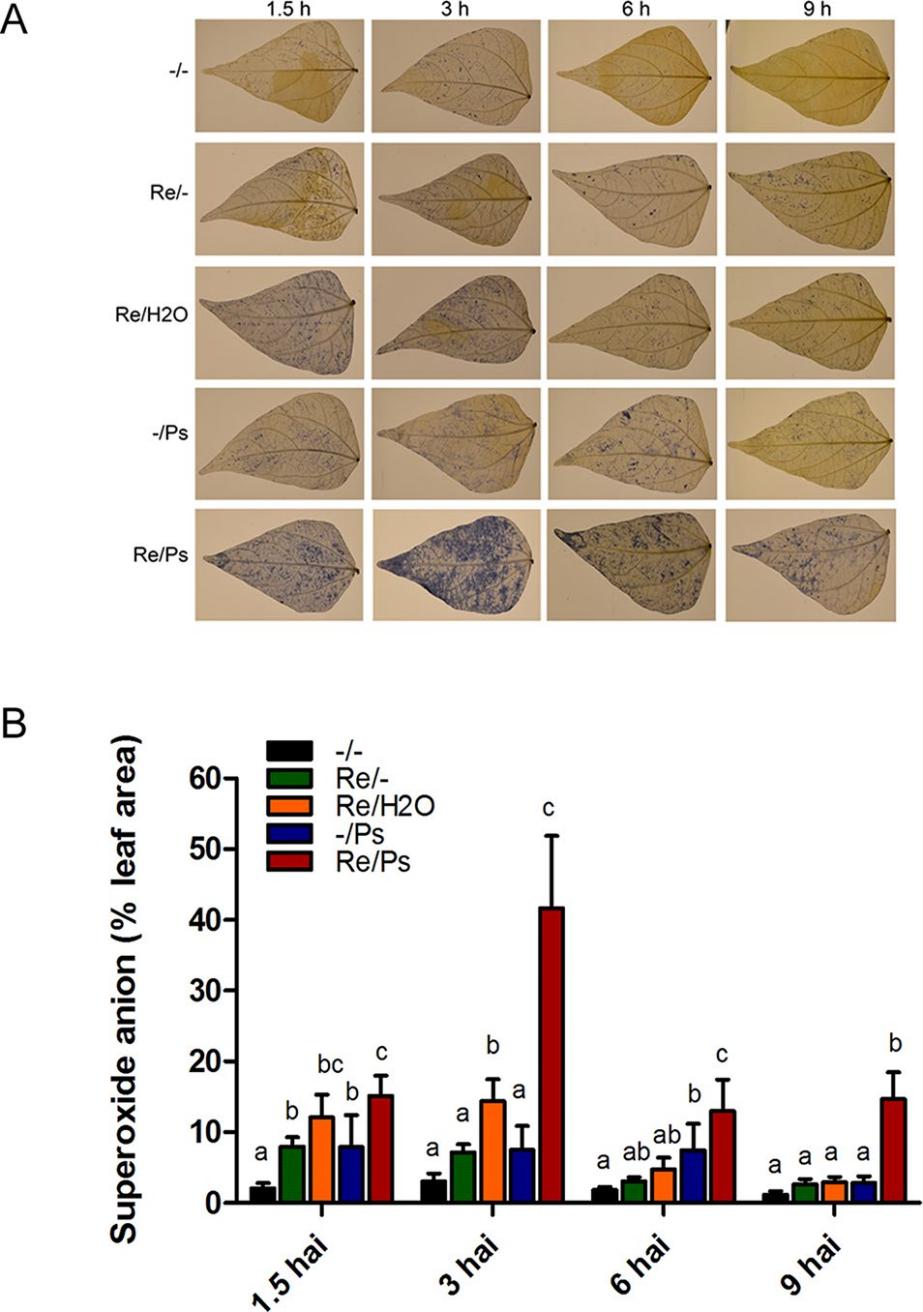
Histochemical detection of superoxide anion in F0 common bean plants at 1.5, 3, 6, and 9 h after infection (hai). **(A)** Illustrative photographs from three independent experiments on *Phaseolus vulgaris* leaves from plants co-cultivated or not with *Rhizobium etli*, after exposure to PspNPS3121. **(B)** Quantification of O_2_^−^ was performed using the protocol described by Juszczak and Baier, 2014. Images were taken using a digital camera Nikon 5500 (Nikon Corp., Tokyo, Japan). Statistical significance was determined using one-way ANOVA followed by Tukey’s test, p < 0.05. Means with the same letter are not significantly different.

Given that infection development induces oxidative stress, we performed a fast and efficient histochemical assay to examine activation of the induced resistance and to detect accumulation of the reactive oxygen species (ROS) superoxide anion (O_2_^−^) in common bean plants at 1.5, 3, 6, and 9 h after PspNPS3121 infection. Plants inoculated with *R. etli* and exposed to the pathogen accumulated 3–4 times more O_2_^−^ than control plants (**Figure 2**), and a high accumulation of O_2_^−^ was observed 3 h after infection at the whole-leaf level. The above indicates that treatment with *R. etli* primes common bean plants for improved induction of certain cellular defense responses.

### Effect of *R. etli* Inoculation on Plant Height, Nodule Number and Size, and Acetylene Reduction After Pathogen Infection

**Figure 3:**
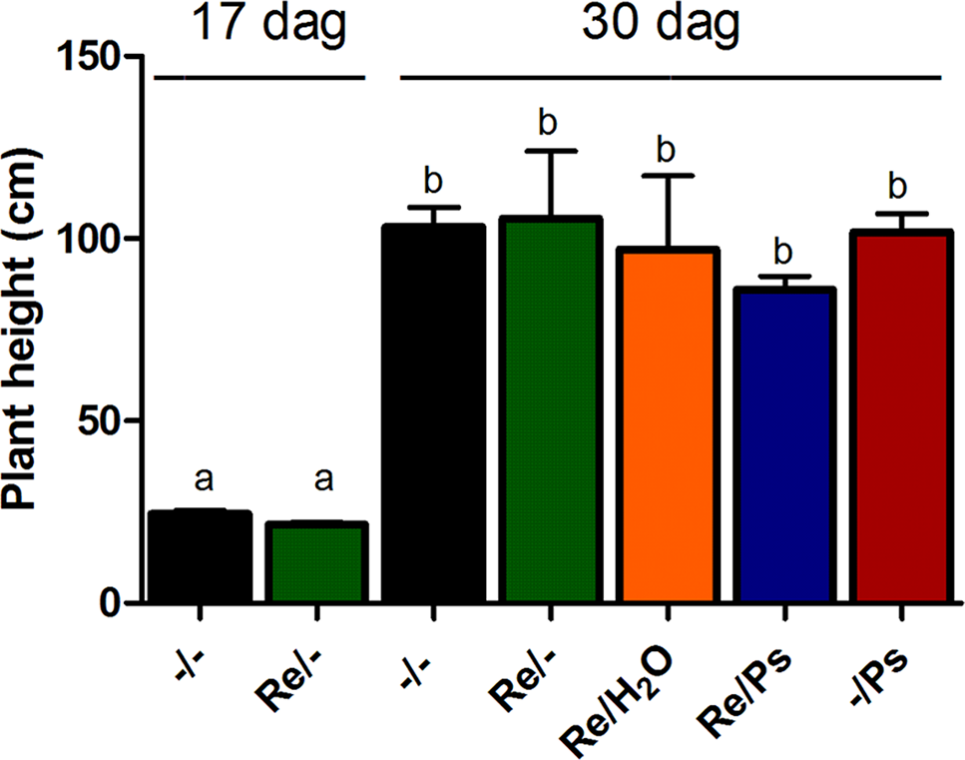
Plant height before pathogen inoculation and 13 days after infection (17 and 30 dag, respectively). Data are mean ± SEM from 24 independent (n = 24) plants per treatment, from two independent experiments, and were analyzed with one-way ANOVA followed by Tukey’s multiple comparison tests (P < 0.05). Means with the same letter are not significantly different.

Plant height in all treatments was quantified before pathogen inoculation and 13 days after infection (17 and 30 dag, respectively; **Figure 3**) by measuring the length of the shoot systems from 24 plants per treatment. Plants inoculated with *R. etli* and exposed to PsNPS3121 were similar in size to plants that were only inoculated with the symbiont, or to the control plants. There were no significant differences in plant height after 13 dpi.

**Figure 4:**
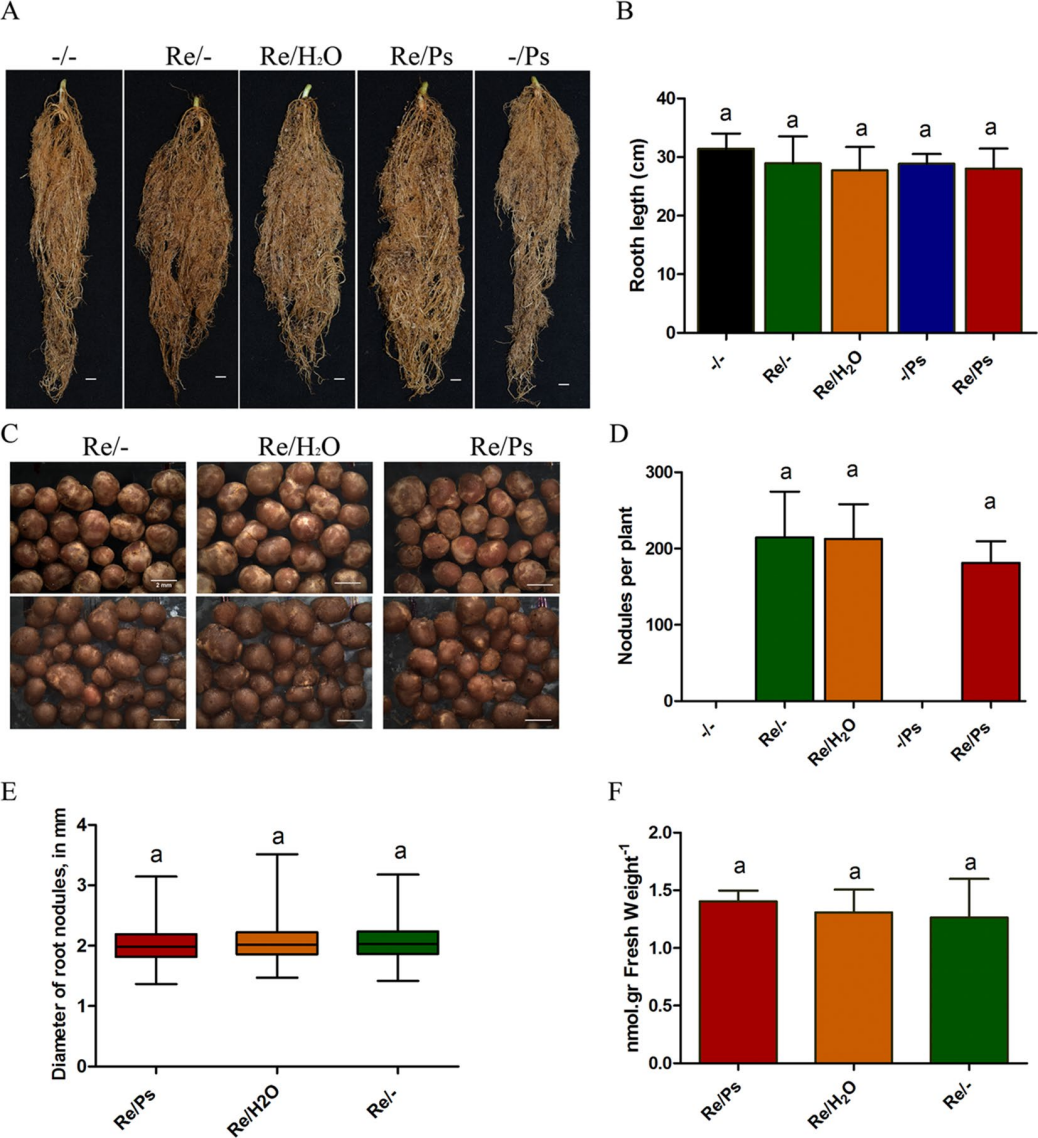
Effects of *Rhizobium etli* and PsNPS3121 on roots and nodules of *Phaseolus vulgaris*. **(A**, **B)** Lengths of the root systems. Data are mean ± SD from 12 independent plants (n = 12). **(C**, **D)** Total number of nodules from each of the different treatments: plants inoculated with *R*. *etli* alone or *R*. *etli* plus PsNPS3121. Data are mean ± SD from nine independent plants (n = 9). **(E)** Diameters of nodules in mm. Data are mean ± SD from 50 nodules in nine independent plants (n = 450). Results are expressed as means and quartiles (box plots). **(F)** Acetylene reduction assay. Data are mean ± SD from six independent plants (n = 6). Statistical significance was determined using one-way ANOVA followed by Tukey’s test, p < 0.05. Means with the same letter are not significantly different.

Subsequently, at 13 dpi with PsNPS3121 (or 30 dag), the plants were extracted, the soil removed, and the lengths of the root systems measured. Root system lengths were not influenced by the different treatments (**Figure 4A–B**). In addition, we counted the total number of nodules from six plants from each of the different treatments: (i) *R. etli* plus PsNPS3121 (Re/Ps), (ii) *R. etli* plus water (Re/H_2_O), and (iii) *R. etli* alone (Re/-). This experiment showed that the numbers of nodules among different treatments are not significantly different (**Figure 4C-D**). We further analyzed the effect of PsNPS3121 on nodule formation by measuring nodule diameter in six plants in each of the different treatments. Nodule diameters from PsNPS3121 infected plants were not significantly different compared to control nodules (Re/H_2_O and Re/-; **Figure 4E**).

Subsequently, we evaluated nitrogen fixing activity in nodules. By measuring nitrogen fixation (nitrogenase activity), *via* an acetylene reduction assay, we observed that all plants from the Re/Ps treatment had capacity for nitrogen fixation similar to the control nodulated roots (**Figure 4F**).

### *R. etli* Prime Plants for Enhanced Expression of Defense Related Genes

Next, we performed a methodical analysis to select illustrative common bean genes, based on their involvement in plant defense and priming, for their expression analysis by qPCR (according to Mayo et al., 2016). After conducting an exhaustive literature review, we selected and identified the corresponding genes at the NCBI and Phytozome databases. Based on transcriptomic data available at the Phytozome, several genes were selected according to their expression patterns (genes with low or no expression in leaves were eliminated). After we confirmed their expression in common bean leaves, three types of genes were selected: (a) genes highly expressed in leaves, (b) genes exhibiting medium expression levels in leaves, and (c) genes exhibiting low expression levels in leaves (**Table 2**).

**Table 2:** Genes selected for defense response and priming, and their experimental expression during ISR in *Phaseolus vulgaris* leaves.

Gene	Reference	Accession Number NCBI	Phytozome Id	Functional annotation at Phytozome	Expression level in common bean leaves
*ERF6*	Huang et al., 2016; Sewelam et al., 2013	XP_007157260.1	*Phvul.002G055800*	EREBP-like factor (EREBP)	Low
*PR10*	Gao et al., 2012	XP_007159109.1	*Phvul.002G209500*	Pathogenesis-related protein Bet v I family (Bet_v_1)	Low
*MYC2*	Pozo et al., 2008; Kazan and Manners, 2013	XP_007156435.1	*Phvul.003G285700*	Transcription factor MYC2	Medium
*PR1*	Zimmerli et al., 2000; Martinez-Aguilar et al., 2016; Slaughter et al., 2012; Luna et al., 2014	XP_007148301.1	*Phvul.006G196900*	Pathogenesis-related protein 1 (PR1)	Medium
*WRKY29*	Martínez-Aguilar et al., 2016; Singh et al., 2014	XP_007160109.1	*Phvul.002G293200*	WRKY DNA-binding domain (WRKY)	Medium
*WRKY70*	Alvarez-Venegas et al., 2007; Luna et al., 2014	XP_007136415.1	*Phvul.009G043100*	WRKY DNA-binding domain (WRKY)	Medium
*PAL2*	Kohler et al., 2002	XP_007144370.1	*Phvul.007G150500*	Phenylalanine ammonia lyase	High
*WRKY33*	Zheng et al., 2006; Birkenbihl et al., 2012	XP_007142085.1	*Phvul.008G251300*	WRKY transcription factor 33 (WRKY33)	High
*NPR1*	Kohler et al., 2002; Van der Ent et al., 2009a; Van der Ent et al., 2009b; Luna et al., 2012	XP_007147518.1	*Phvul.006G131400*	Regulatory protein NPR3related	High

**Figure 5:**
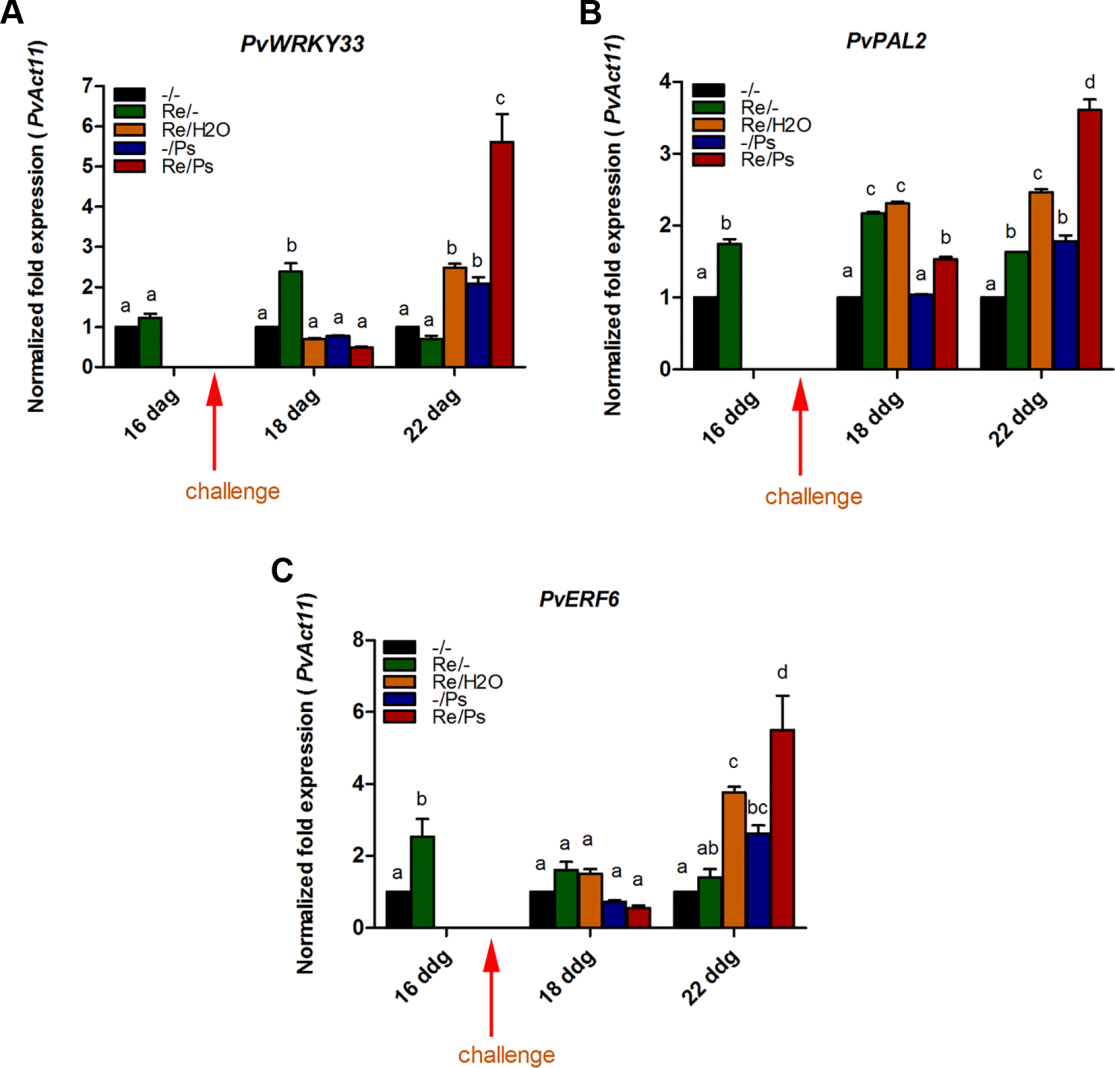
Transcript levels of genes involved in plant defense. **(A)** PvWRKY33; **(B)** PvPAL2; **(C)** PvERF6. Plants were primed with R. etli, followed by inoculation with PspNPS3121 (Re/Ps), inoculated only with the symbiont (Re/- ; and water, Re/H2O), infected only (-/Ps), or neither primed nor inoculated (control, -/-). Data were normalized to the Actin11 (PvActin11) and Tubulin (PvTUB, see **Supplementary Figure SF1**) reference genes. Data represent mean ± SD from three independent experiments (n = 3). Statistical significance was determined using two-way ANOVA followed by Tukey’s multiple comparison test, p < 0.05. Dag: days after germination. Means with the same letter are not significantly different.

Accordingly, we examined the effect of *R. etli* inoculation (priming) on the transcription levels of nine genes related to ISR and defense and potentially involved in priming. Samples were obtained from plants treated with *R. etli* (Re/-) only, with the pathogen (Ps/-) only, or during the interaction with *R. etli* and exposed to the pathogen (Re/Ps). After qPCR analysis, the transcript levels of only three genes from plants that had been primed with *R. etli* and inoculated with PspNPS3121 showed a transcriptional pattern characteristic of the priming response (e.g., biphasic curve). In systemically resistant leaves of *R. etli*–treated plants, priming alone did not enhance transcription of *PvWRKY33, PvERF6*, and *PvPAL2*. However, 120 h after pathogen inoculation, there was high accumulation of transcripts, compared to the control plants (**Figure 5** and **Supplementary Figure SF1**). WRKY33 is a transcription factor (TF) involved in the activation of the salicylic acid (SA)–related host response (Birkenbihl et al., 2012); ERF6 is a TF that activates expression of PR genes (Huang et al., 2016), whereas PAL2 functions in plant secondary metabolites synthesis and callose deposition (Kohler et al., 2002). Consequently, *R. etli* primed *P*. *vulgaris* plants for potentiated gene activation, which was subsequently induced by PspNPS3121 infiltration.

On the contrary, there was enhanced transcription of *PvPR1* in the late stages of priming (120 h after bacterial inoculation; **Supplementary Figure SF2**). Such a transcriptional pattern, however, was also induced by the pathogen alone. Therefore, the rest of the genes could not be considered as having been primed, under the present experimental conditions.

### *R. Etli* Activates the Expression of Genes Involved Plant Hormone Biosynthesis

**Figure 6:**
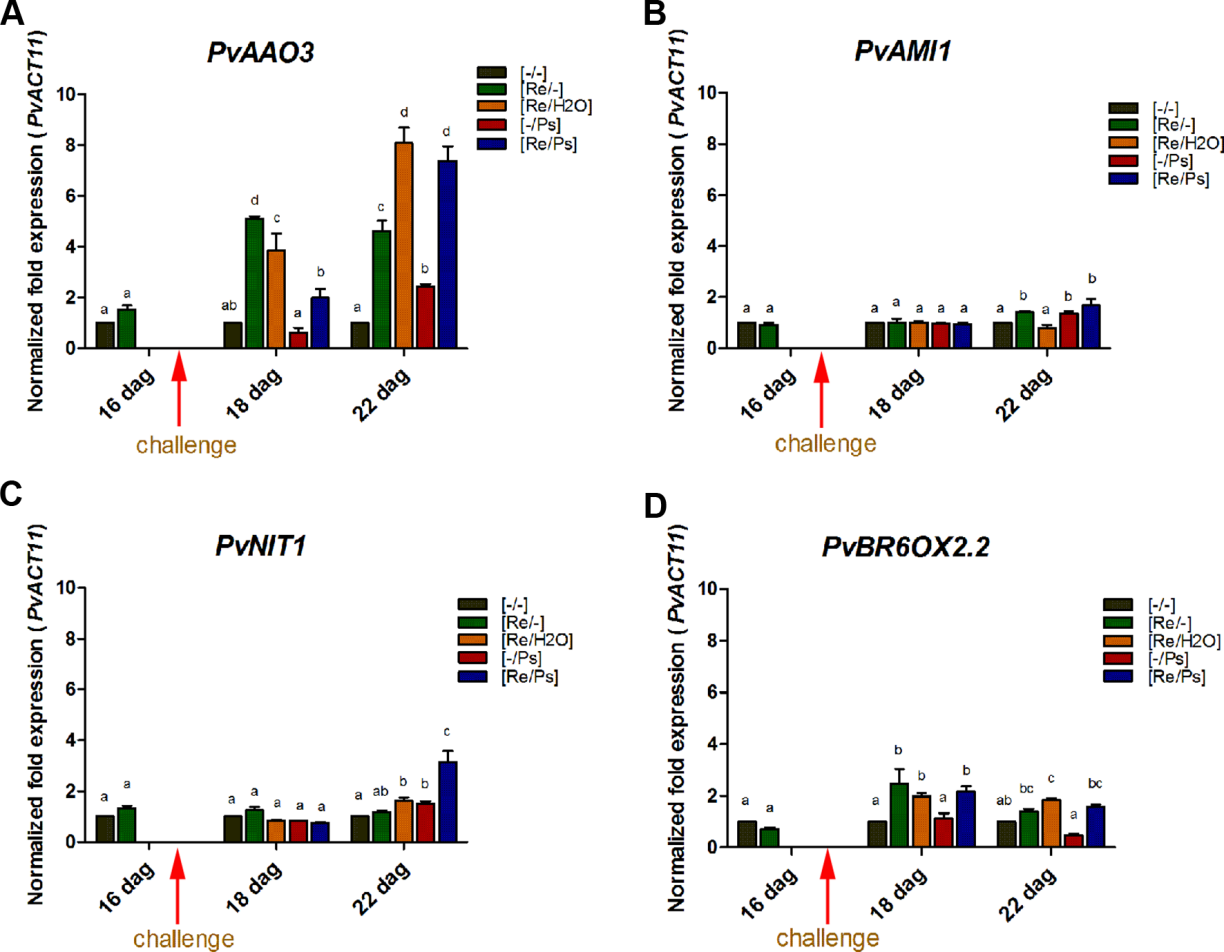
Transcript levels of genes involved in plant hormone biosynthesis. **(A)** PvAAO3; **(B)** PvAMI1; **(C)** PvNIT1; **(D)** PvBROX2.2. Plants were primed with R. etli, followed by inoculation with PspNPS3121 (Re/Ps), inoculated only with the symbiont (Re/- ; and water, Re/H2O), infected only (-/Ps), or neither primed nor inoculated (control, -/-). Data were normalized to the Actin11 (PvActin11) and Tubulin (PvTUB, see **Supplementary Figure SF3**) reference genes. Data represent mean ± SD from three independent experiments (n = 3). Statistical significance was determined using two-way ANOVA followed by Tukey’s multiple comparison test, p < 0.05. Dag: days after germination. Means with the same letter are not significantly different.

Plant hormones regulate numerous developmental processes and signaling networks involved in plant responses to different types of biotic and abiotic stresses (Bari and Jones, 2009). Furthermore, the study of key genes and enzymes involved in plant hormone biosynthesis provides important information concerning the regulation of plant hormones pathways and can offer new information regarding the function of plant hormones during plant-microbe interactions (Seo et al., 2004). Thus, we determined the transcript levels of some key genes involved in the synthesis of auxins (IAA; involved in the modulation of defense and development responses; Bari and Jones, 2009), ABA (involved in biotic stress; Ton and Mauch-Mani, 2004; Ton et al., 2009), and BR (implicated in induced systemic tolerance to biotic stress and in the modulation of plant defense responses; Bari and Jones, 2009; Li et al., 2013) (**Figure 6** and **Supplementary Table ST1**). Specifically, we determined the transcript levels of the *AMI1* (*INDOLE-3-ACETAMIDE HYDROLASE 1*), *NIT1* (*NITRILASE 1*), *AAO3* (*ABSCISIC ALDEHYDE OXIDASE 3*), and *BR6OX2.2* (*BRASSINOSTEROID-6-OXIDASE 2 ISOFORM 2*). Compared to control plants (**Figure 6** and **Supplementary Figure SF3**), the transcripts of *PvAAO3* (which codes for an enzyme that catalyzes the last step in the ABA biosynthesis pathway, from abscisic aldehyde to ABA; Koiwai et al., 2004; Seo et al., 2004) and *PvBR6OX2.2* (enzyme that catalyzes the last step in the production of brassinolide; Kim et al., 2005) increased in all treatments where the plants were inoculated with *R. etli*. As shown in **Figure 6**, there was an induction in *PvAAO3* and *PvBR6OX2.2* transcripts in all three samples that were inoculated with *R. etli* (Re/-, Re/H_2_O, Re/Ps). In control plants (-/-) or in plants treated with the pathogen only (-/Ps), however, the transcript levels of *PvAAO3* and *PvBR6OX2.2* remained very low. Intriguingly, infiltration with water (Re/H_2_O), after *R. etli* inoculation, resulted in similar *PvAAO3* and *PvBR6OX2.2* transcript levels to the Re/Ps treatment (at 24 h and 120 h after PspNPS3121 infection, respectively), indicating that their induction was potentiated by treatment with water. This result suggests that *R. etli* inoculation enhances transcription of *PvAAO3* and *PvBR6OX2.2*, which in turn could increase ABA and brassinolide synthesis, independently of PspNPS3121 infection. The rest of the genes analyzed (*PvNIT1*, *PvAMI1*), involved in auxin biosynthesis, did not show significant changes in their transcripts as a result of the different treatments.

### *R. etli*–Induced Callose Deposition in *P. vulgaris*

**Figure 7:**
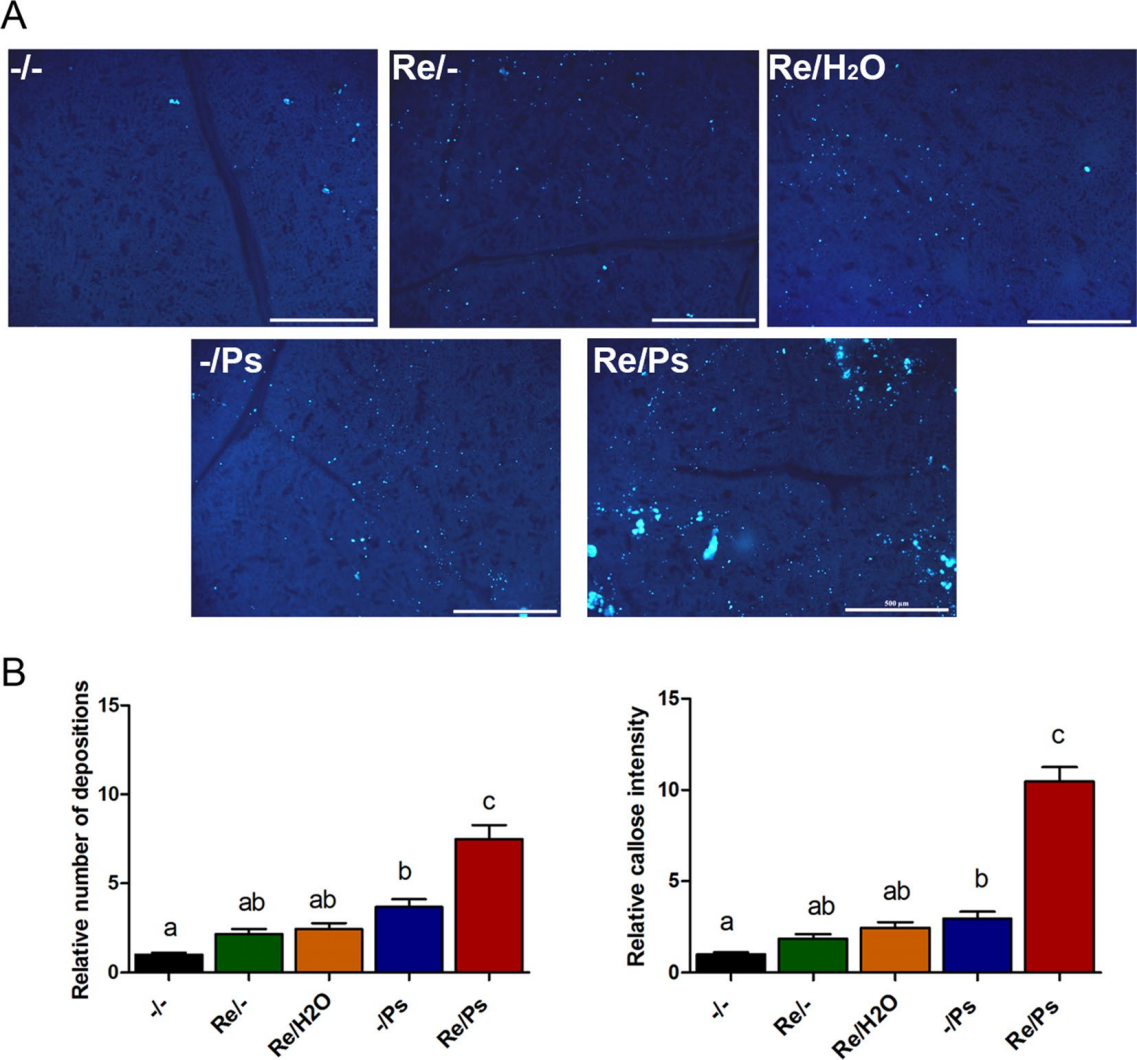
Phenotype of R. etli-induced callose in F0 generation. **(A)** Morphological differences of callose depositions in 17 days-old P.vulgaris plants (co-cultivated with the symbiont), after PspNPS3121 infection. Representative photographs of aniline blue-stained leaves under UV epifluorescence illustrate differences in callose deposition between treatments. **(B)** Relative quantification of callose (number of individual depositions per unit of leaf surface) and callose intensity (number of fluorescent callose-corresponding pixels relative to the total number of pixels) were determined by using the Fiji software (according to Jin and Mackey, 2017). Values represent means (±SEM, n=24). Statistical significance was determined using one-way ANOVA followed by Tukey’s multiple comparison test, p < 0.05). Scale bar = 500 µm. Means with the same letter are not significantly different.

To quantify activity of plant defense response, we determined callose deposition (a quick cellular defense outcome; Kohler et al., 2002), in response to *R. etli* inoculation and to PspNPS3121 infection. After bacterial treatment, common bean leaves were stained with aniline blue analyzed by epifluorescence microscopy, and callose was quantified and documented as the “relative number of callose-corresponding pixels (callose intensity) or the relative number of callose depositions” (Luna et al., 2011). As shown in **Figure 7**, plants primed with *R. etli* and inoculated with PspNPS3121 (Re/Ps) showed an increase in the number of callose depositions and callose intensity compared to the rest of the different treatments (Re/H_2_O; -/Ps; Re/-), depicting the primed callose response. Furthermore, the size of the individual callose depositions was larger in Re/Ps treated plants, indicating that *R. etli* enhanced the production of callose and generated greater amounts of callose per deposition, which was induced by PspNPS3121 infection.

The number of depositions and callose intensity between control plants (-/-) and plants inoculated only with the symbiont (Re/-; Re/H_2_O) did not differ statistically. Whereas the relative number of depositions and intensity did not differ statistically between plants inoculated only with the symbiont (Re/-; Re/H_2_O) and plants infected only with the pathogen (-/Ps).

### Transgenerational Inheritance of Priming in the Common Bean

To evaluate if *R. etli*–primed plants had the potential to generate an transgenerational memory and produce offspring that are more resistant to the halo blight disease than progeny of unprimed parents, all plants (F0 generation), from all the different treatments (-/-, Re/-, Re/H_2_O, -/Ps, and Re/Ps), were self-pollinated and grown to seed set to generate the F1 progeny.

**Figure 8:**
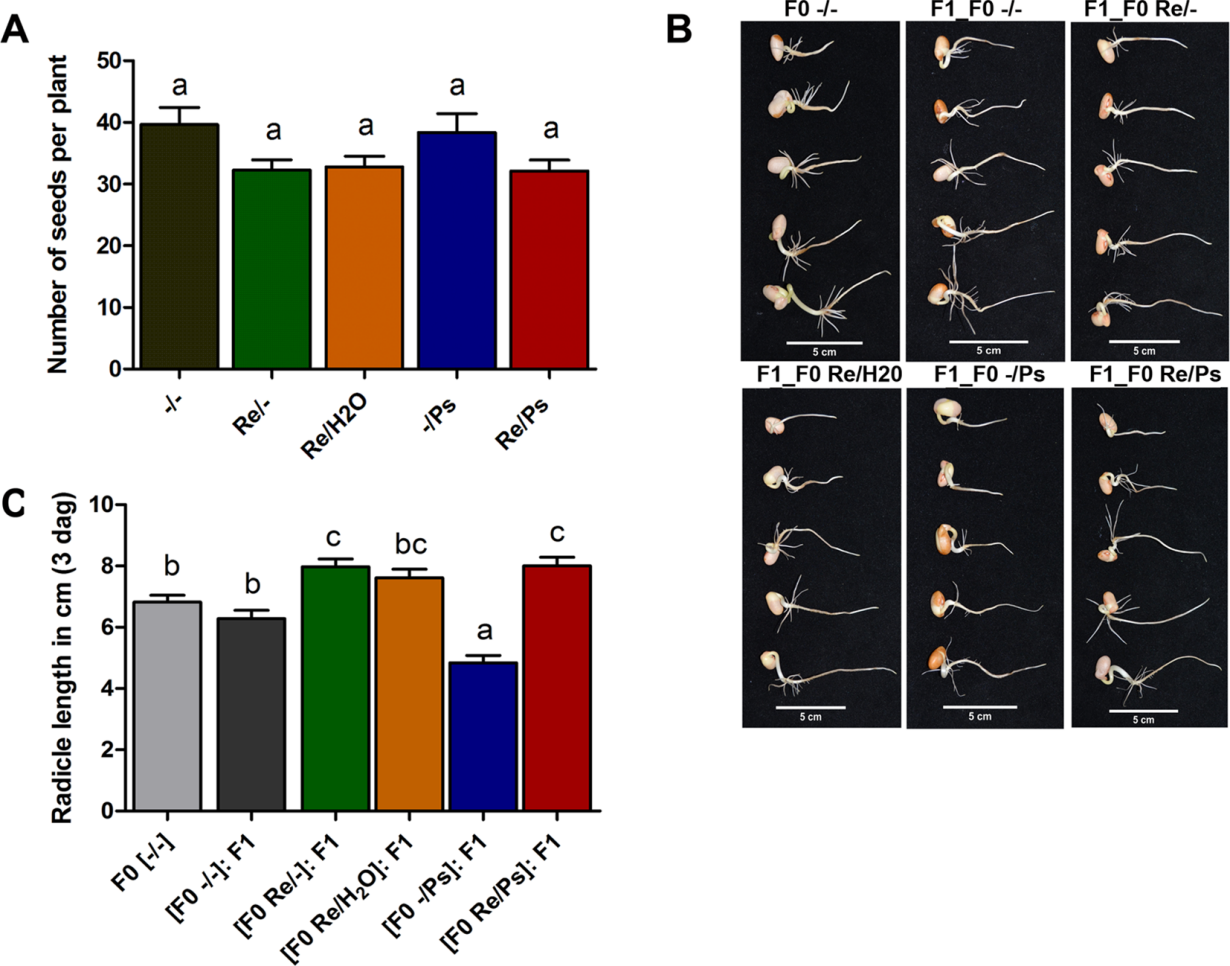
**(A)** Number of seeds produced in plants from the different treatments (Re/Ps, Re/H2O, Re/-; -/Ps, and -/-). Data are mean ± SD from six independent plants (n = 6) per experiment, from three independent experiments. **(B-C)** Radicle length of F1 seedlings three days after germination. Scale = 5 cm. Data are mean ± SD from 75 independent seeds (n = 75), from three independent experiments. Statistical significance was determined using one-way ANOVA followed by Tukey’s multiple comparison test, p < 0.05. Means with the same letter are not significantly different.

As shown in **Figure 8A**, there was no significant difference in the number of seeds produced by plants treated with *R. etli* (Re/-, Re/H_2_O, Re/Ps) and the number of seeds produced by control plants (-/-) or plants treated with the pathogen only (-/Ps). However, there was a marginal difference between plants that were nitrogen-supplemented (-/- and -/Ps) and plants that were fertilized with a nitrogen-free solution (Re/-, Re/H_2_O, Re/Ps). The results suggest that *R. etli* directly or indirectly protected its hosts against pathogen attack, and its presence facilitates the maintenance of proper crop yield.

Next, we germinated F1 seeds from all the different F0 treatments (-/-, Re/-, Re/H_2_O, -/Ps, and Re/Ps) in germination trays under sterile conditions, and the lengths of the radicles were measured 3 dag, before transferring the seedlings to pots. There were significant differences in radicle length in F1 seedlings when compared with F0 control seedlings (**Figure 8B, C**). Particularly, seedlings from plants that were treated with the pathogen only in the F0 generation were 29% shorter than the untreated control plants. Notably, seedlings from plants that were treated with the symbiont in the F0 [(F0 Re/Ps): F1; (F0 Re/-): F1; **Figure 8C**] had radicles that were 17% longer than the radicles in the control plants (F0 -/-).

**Figure 9:**
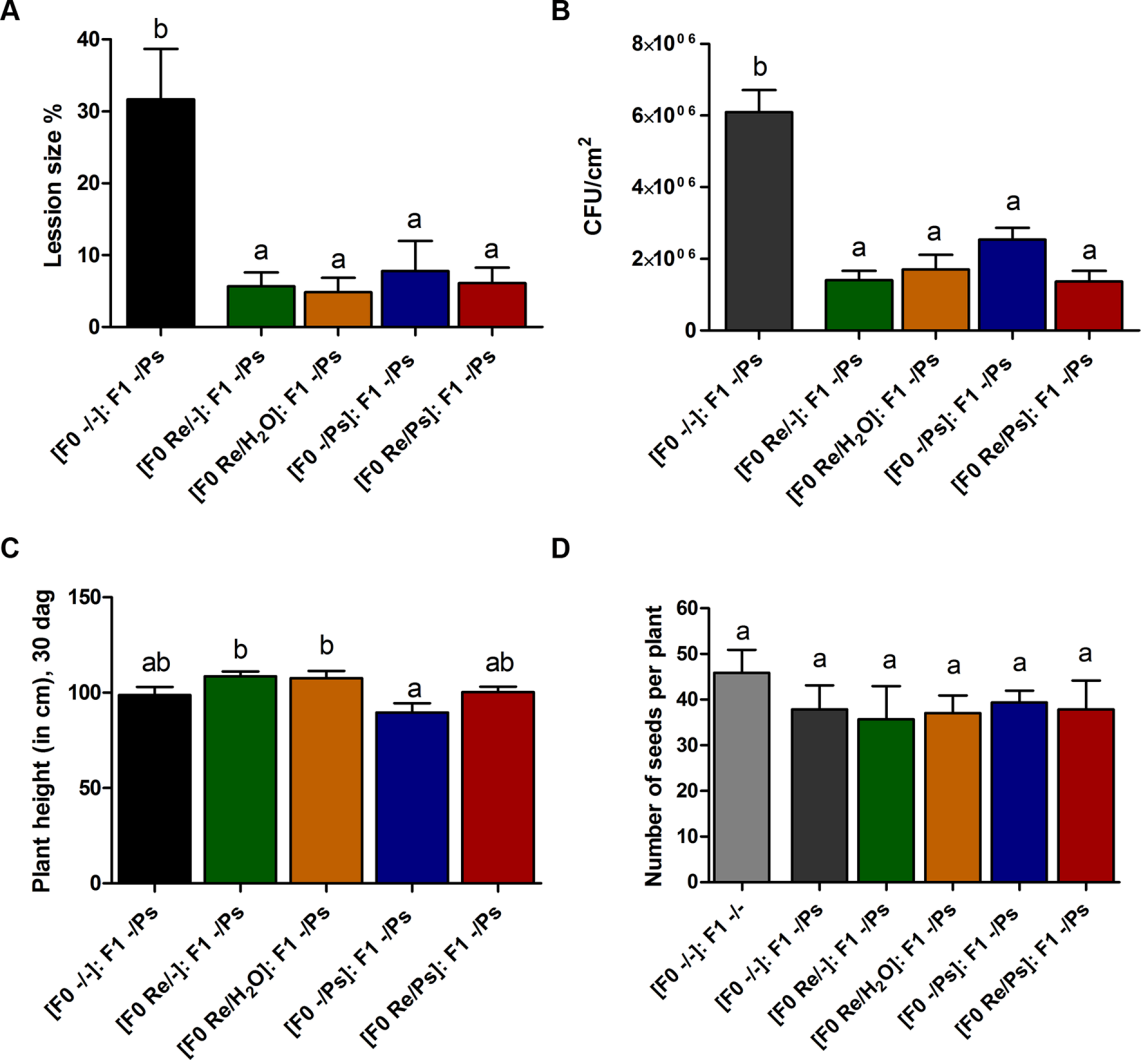
Transgenerational priming effect. **(A)** Lesion size and **(B)** colony forming units (CFU) in F1 common bean plants 10 days after infection with PspNPS3121. Data are mean ± SEM from three independent experiments. **(C)** Plant height 13 days after infection (30 dag). Data are mean ± SEM from 24 independent (n = 24) plants per treatment, from two independent experiments. **(D)** Number of seeds produced in F1 plants from the different treatments (Re/Ps, Re/H_2_O, Re/-; -/Ps, and -/-). Data are mean ± SD from six independent plants (n = 6) per experiment, from three independent experiments. Statistical significance was determined using one-way ANOVA followed by Tukey’s multiple comparison test, p < 0.05. Means with the same letter are not significantly different.

At 17 dag, two trifoliate leaves from all F1 plants were exposed to PspNPS3121 [(F0 Re/Ps): F1 -/Ps; (F0 Re/H_2_O): F1 -/Ps; (F0 Re/-): F1 -/Ps; (F0 -/Ps): F1 -/Ps]. Treatment with the pathogen resulted in a 5–8% lesion size (of total leaf area), among all plants—that is, an 85% reduction in lesion size when compared to the control plants [(F0 -/-): F1 -/Ps] (**Figure 9A**). In addition, PspNPS3121 bacterial populations (CFU) in leaves from plants treated with *R. etli* in the F0 generation and exposed to the pathogen in the F1 generation [(F0 Re/Ps): F1 -/Ps; (F0 Re/H_2_O): F1 -/Ps; (F0 Re/-): F1 -/Ps] were significantly lower than in the control plants treated with the pathogen only ([F0 -/-]: F1 -/Ps) (**Figure 9B**). Therefore, common bean plants treated with *R. etli* in the F0 generation were effectively protected in the F1 generation against PspNPS3121, in contrast to plants that were not primed [(F0 -/-): F1 -/Ps]. Moreover, F0 plants that were treated with the pathogen only (“sensitized”; Conrath, 2009) and exposed to the pathogen in the F1 generation [(F0 -/Ps): F1 -/Ps] also have shown lower CFUs compared to the control plants (**Figure 9B**). We inferred that infection with PspNPS3121, in the F0 generation, induced enhanced pathogen defense (priming effect) in the F1 generation against the halo blight bacterial disease. However, continuous exposure to the pathogen [(F0 -/Ps): F1 -/Ps] influenced plant height when compared to plants that were primed with *R. etli* in the F0 generation (**Figure 9C**). That is, plants exposed only to the pathogen in F0 and F1 [(F0 -/Ps); (F0 -/Ps): F1 -/Ps; **Figure 9C**] were 17% shorter 30 dag than plants inoculated with the symbiont [(F0 Re/H_2_O): F1 -/Ps; (F0 Re/-): F1 -/Ps; **Figure 9C**]. However, there was no significant difference in the number of seeds produced between F1 plants treated PspNPS3121 and the control plants (-/-) (**Figure 9D**).

**Figure 10:**
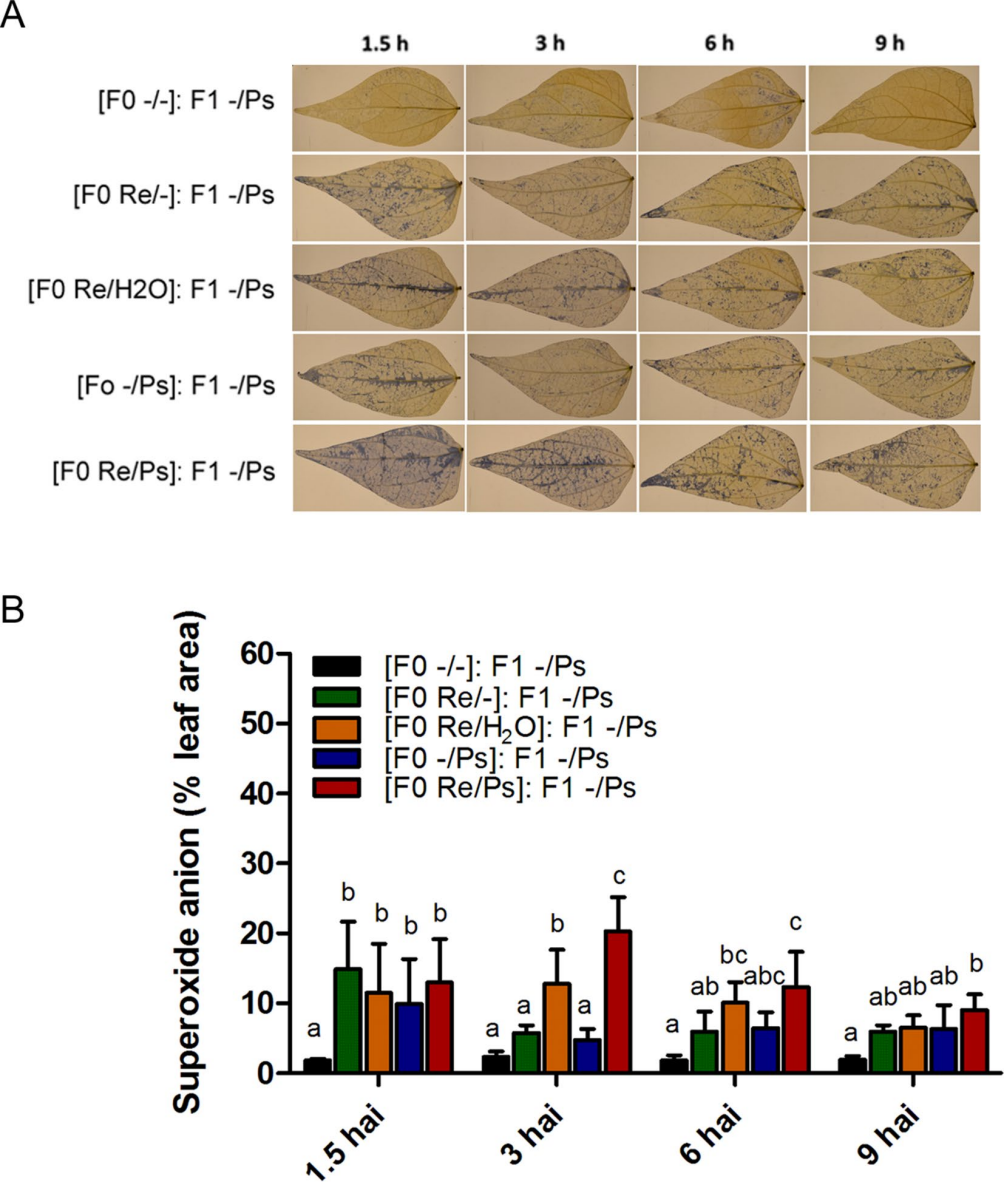
Histochemical detection of superoxide anion in F1 common bean plants at 1.5, 3, 6, and 9 h after infection. Seeds from all F0 plants (-/-, Re/-, Re/H_2_O, -/Ps, and Re/Ps) were germinated and F1 progeny plants were challenged with the pathogen. **(A)** Illustrative photographs from three independent experiments on *Phaseolus vulgaris* leaves from F1 plants after exposure to PspNPS3121. **(B)** Quantification of O_2_^−^ was performed using the protocol described by Juszczak and Baier, 2014. Images were taken using a digital camera Nikon 5500 (Nikon Corp., Tokyo, Japan). Statistical significance was determined using one-way ANOVA followed by Tukey’s test, p < 0.05. Means with the same letter are not significantly different.

We next wished to examine the activation of the induced resistance in F1 plants by detecting accumulation of the O_2_^−^ at 1.5, 3, 6, and 9 h after PspNPS3121 infection (**Figure 10**). All plants from the F0 generation, when exposed to the pathogen in the F1 generation, accumulated more O_2_^−^ than control plants [(F0 -/-): F1 -/Ps], at 1.5 h after infection. However, higher accumulation of O_2_^−^ was observed 3 h after infection at the whole-leaf level, in leaves from plants treated with *R. etli* and PspNPS3121 in the F0 generation and exposed to the pathogen in the F1 generation [(F0 Re/Ps): F1 -/Ps], as well as in plants treated with *R. etli* and water [(F0 Re/H_2_O): F1 -/Ps], when compared to control plants (**Figures 7A, B**). Such O_2_^−^ accumulation pattern suggests a transgenerational effect in plants primed in the F0 generation with *R. etli* (“bacterizing the roots,” or root colonized by bacteria; Van Peer et al., 1991) and induced in the F1 by PspNPS3121 on cellular defense responses.

**Figure 11:**
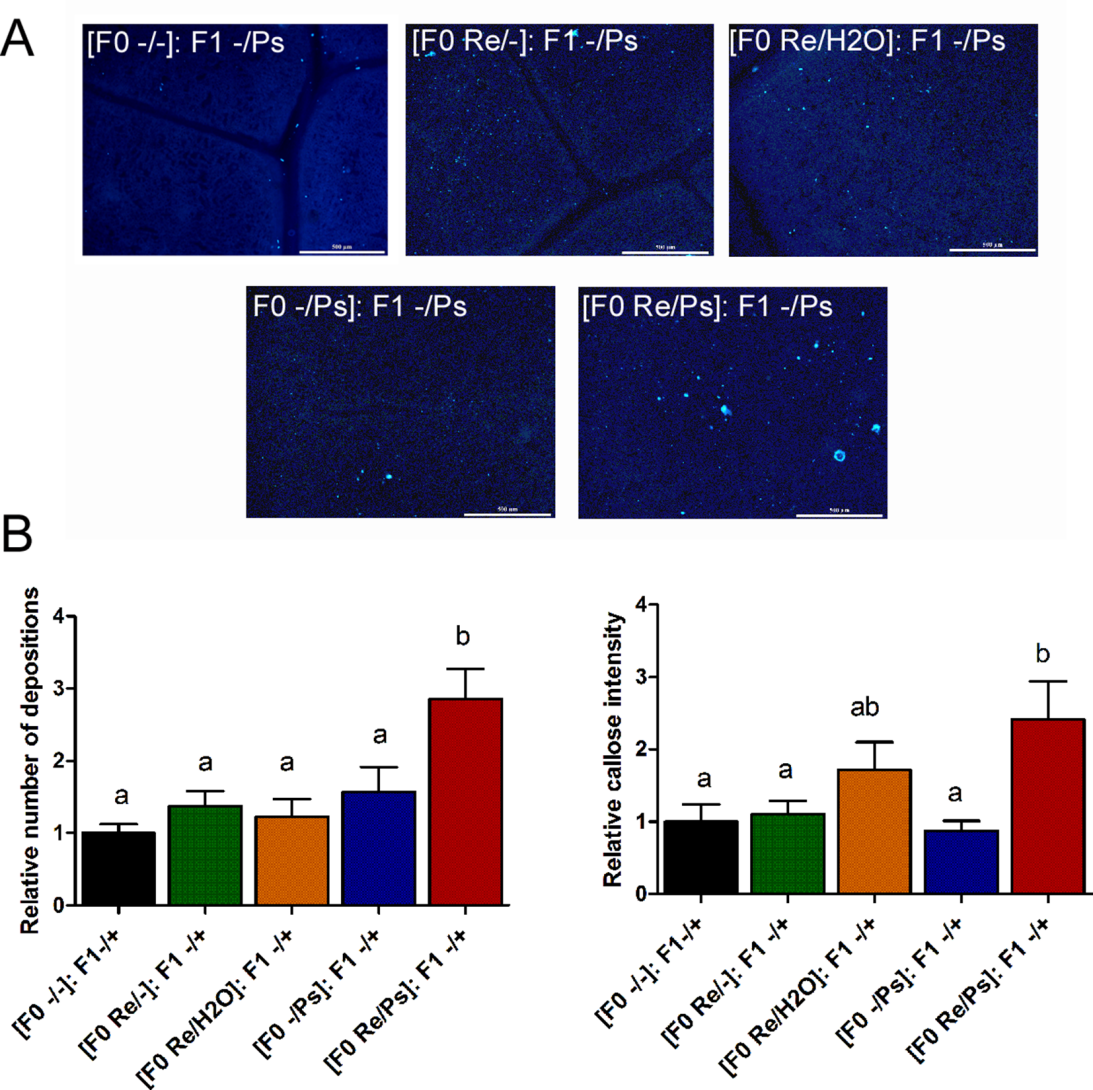
Phenotype of R. etli-induced callose in F1 generation. **(A)** Morphological differences of callose depositions in 17 days-old P.vulgaris F1 plants. Seeds from the F1 progenies were germinated and exposed to the pathogen only, without rhizobacteria treatment. Representative photographs of aniline blue-stained leaves under UV epifluorescence illustrate differences in callose deposition between treatments. **(B)** Relative quantification of callose (number of individual depositions per unit of leaf surface) and callose intensity (number of fluorescent callose-corresponding pixels relative to the total number of pixels) were determined by using the Fiji software (according to Jin and Mackey, 2017). Values represent means (±SEM, n=24). Statistical significance was determined using one-way ANOVA followed by Tukey’s multiple comparison test, p < 0.05). Scale bar = 500 µm. Means with the same letter are not significantly different.

In addition, to determine whether the primed callose response has a transgenerational effect against PspNPS3121, we analyzed callose depositions in the F1 generation of all the different treatments, after exposure to the pathogen [(F0 -/-): F1 -/Ps; (F0 -/Ps): F1 -/Ps; (F0 Re/H_2_O): F1 -/Ps; (F0 Re/-): F1 -/Ps; (F0 Re/Ps): F1 -/Ps]. As shown in **Figure 11**, plants treated with *R. etli* and PspNPS3121 in the F0 generation and exposed to the pathogen in the F1 generation [(F0 Re/Ps): F1 -/Ps] showed a faster and stronger accumulation of callose depositions after pathogen infection, even though the relative number of depositions was 2.6 times lower than in the F0 generation. This suggests that the ability to produce callose in F1 is caused by the priming effect in the F0. Plants from the rest of the treatments, however, failed to accumulate enhanced levels of callose after pathogen treatment.

**Figure 12:**
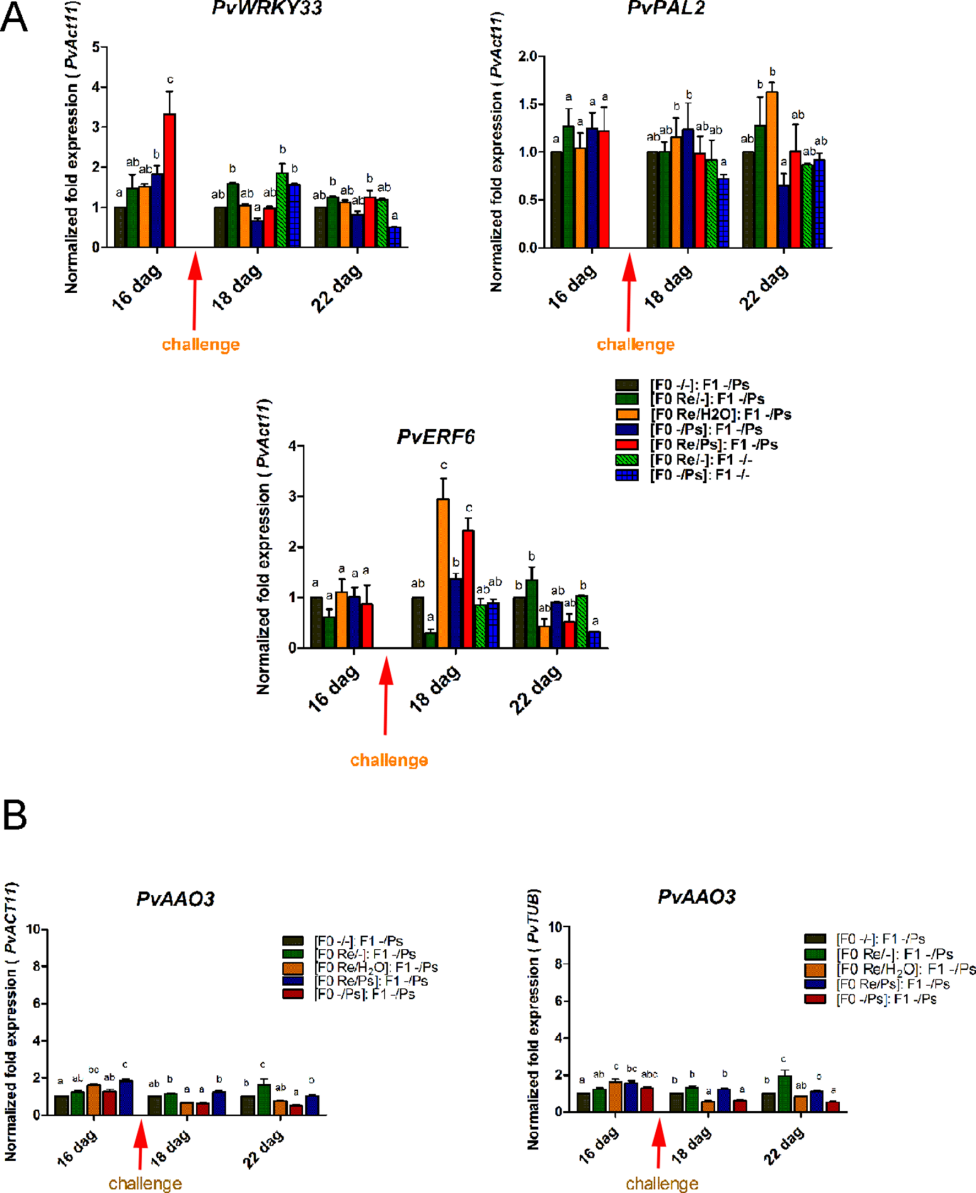
Transgenerational transcript levels of common bean genes. **(A)** Transcript levels of genes involved in plant defense. **(A)** *PvWRKY33*; **(B)** *PvPAL2*; **(C)** *PvERF6*. F0 plants were self-pollinated and grown to seed set to generate the F1 progeny. Seeds from the F1 progenies were germinated and exposed to the pathogen only, without rhizobacteria treatment. Samples were obtained at 16, 18, and 22 dag. Data were normalized to the actin11 (*PvActin11*) and tubulin (*PvTUB*, see **Figure SF4**) reference genes. **(B)** Transcript levels of the *PvAAO3* gene involved in ABA synthesis. Data were normalized to the actin11 (*PvActin11*) and tubulin (*PvTUB*) reference genes. Data are mean ± SD from three independent experiments (n = 3). Statistical significance was determined using two-way ANOVA followed by Tukey’s multiple comparison test, p < 0.05. Dag, days after germination.

We then selected the primed-responsive genes (*PvWRKY33, PvPAL2,* and *PvERF6*) highly induced against PspNPS3121 in the parental generation (F0) and analyzed their expression patterns in the F1 generation before and after pathogen attacks (**Figure 12** and **Supplementary Figure SF4**). As shown in **Figure 12A**, the genes that exhibited an enhanced transcription pattern following pathogen attack were *PvERF6* (2.5 to three-fold induction, 24 h after infection) and *PvPAL2* (0.5-fold induction, 24 h after infection) when compared to control plants. Consistent with previous results, the finding suggests a transgenerational effect in common bean plants that were primed in the F0 generation with *R. etli* and induced by PspNPS3121 infiltration. In addition, F0 plants treated with *R. etli* were sensitized and displayed enhanced *PvPAL2* and *PvERF6* transcript levels in the F1 generation. However, expression levels of the ABA-synthesis-related gene *PvAAO3*, in the F1 generation (without *R. etli* inoculation), were not significantly different before and after PspNPS3121 infiltrations and also with respect to the control {[(F0 -/-): F1 -/-]; **Figure 12B**}. This suggests that inoculation with *R. etli* is required for *PvAAO3* expression and ABA synthesis and that *PvAAO3* expression is not directly related to the transgenerational induced resistance, under the present experimental conditions.

## Discussion

Common bean is a valuable crop worldwide and an important grain legume in the human diet. Common bean production, however, is affected by numerous pathogens. Some of the major approaches for controlling common bean infectious diseases include crop rotation, sanitation, using certified seeds, growing resistant cultivars, stress and wound avoidance, chemical control or application of pesticides, and to a lesser extent, the use of bacterial biocontrol agents and PGPR. In legumes, the use of PGPR has been mostly limited to rhizobia manipulation in studies aimed at improving legume growth and development, particularly *via* nodulation and nitrogen fixation (Pérez-Montaño et al., 2014). In addition, several studies have examined the hypothesis that PGPR might enable plants to maintain their yield with reduced fertilizer application rates (Yang et al., 2009).

PGPR also suppress disease-causing microbes by activating the ISR plant defense system. In addition, legume-*Rhizobium* interactions induce PGPR “like-responses,” improving tolerance/resistance to different types of stress (Fernandez-Göbel et al., 2019). However, few reports have been published on *Rhizobium* as elicitors of resistance to bacterial diseases (Osdaghi et al., 2011). Therefore, the goals of the present study were, first, to determine whether the symbiont *R. etli* elicits the ISR in *P. vulgaris* against PspNPS3121. Second is to determine if *R. etli* has a role in decreasing or inhibiting the harmful effects of PspNPS3121 on plant growth, development, and nitrogen fixation in *R. etli* treated bean plants. Achievement of the above goals could facilitate the use *R. etli* (and rhizobacteria in general) as a defense-priming agent to induce ISR in *P. vulgaris* to minimize susceptibility to pathogens and enhance plant breeding activities and overall crop productivity. Therefore, we studied the effect of priming on gene activation and the generational effect of the primed state using *R. etli* as an elicitor.

Inoculation of common bean with *R. etli* reduced halo blight severity when compared to plants not treated with the symbiont. For example, foliar pathogen lesion size was 75% lower in the *R. etli* inoculated plants, than in the non-inoculated plants, while PspNPS3121 CFU was 50% lower in *R. etli* inoculated plants. Therefore, the symbiotic relationship between *R. etli* and common bean reduced disease incidence. The finding is notable since any method that can reduce the foliar symptoms of halo blight disease at low costs and in an environmentally safe manner (e.g., without the application of commercial pesticides) is of considerable importance in agriculture.

In our experiments, there were no significant changes in root length, diameter and number of nodules, and nitrogen fixation (nitrogenase activity) among the different treatments. The finding is also critical, since the effects of co-inoculation of *R. etli* and PspNPS3121 on common bean suggest that *R. etli* improves tolerance against halo blight disease without affecting crop productivity. Although further studies are required to corroborate the effectiveness of distinct strains of *Rhizobium* spp. in reducing the incidence of halo blight disease, the utilization of *R. etli* in common bean planting areas is recommended and could be an alternative to the application of synthetic pesticides and fertilizers.

Accordingly, inoculation with *R. etli* elicited ISR and primed the whole plant for enhanced defense against PspNPS3121. Aboveground parts of the plant (foliar tissue) acquired an enhanced level of resistance against infection by the PspNPS3121 pathogen following inoculation with *R. etli*. In addition, *R. etli*–treated plants exhibited considerably higher ROS superoxide anion accumulation at non-toxic levels at the site infected by the pathogen. Moreover, during the different stages of the symbiotic interaction, ROS can be translocated extensively. Therefore, the systemic redox signaling plays an important role in the regulation of systemic acclimatory mechanisms under stress conditions (Fernandez-Göbel et al., 2019). Consequently, ROS signaling networks control a wide range of biological processes, including responses to biotic stimuli, and functions as a general priming signal in plants (Baxter et al., 2014). Moreover, plant defense hormones modulate plants’ ROS status (Sewelam et al., 2013), which suggests that inoculation of *R. etli* to the root system sensitized distal plant parts for enhanced pathogen defense (priming effect). Therefore, long-term responses (Mittler et al., 2011) enhanced the oxidative stress defense capacity in common bean leaves when exposed to the PspNPS3121 pathogen.

Interestingly, our study demonstrates that plants treated with *R. etli* responded more efficiently to pathogen attack *via* an augmented deposition of callose. Thus, *R. etli* sensitizes or primes *P. vulgaris* plants for stronger accumulation of callose biosynthesis at the site of infection, under the present experimental conditions. Hence, we propose that *R. etli*–induced superoxide anion accumulation promotes callose deposition.

*R. etli*–treated plants exhibited a potentiated defense-related gene expression. The transcript levels of *PvWRKY33*, *PvERF6,* and *PvPAL2* exhibited a typical priming response. Although *R. etli* did not trigger their expression, after PspNPS3121 inoculation, transcripts accumulated at levels higher than in the unprimed, inoculated controls. In plants, a large number of *WRKY* genes code for TF induced by pathogen infection are involved in plant defense responses and regulate cross-talk between JA- and SA-regulated disease response pathways (Zheng et al., 2006). In *Arabidopsis*—for example, WRKY33 TF interacts with MAP kinase 4 (MPK4) and MKS1 within the nucleus and upon exposure to PsDC3000 or following elicitation with flg22 (a 22-amino acid sequence of flagellin), WRKY33 is released from the trimeric complex and targets the promoter of *PHYTOALEXIN DEFICIENT3* (*PAD3*), encoding an enzyme necessary for the synthesis of antimicrobial camalexin, a phytoalexin with antimicrobial and antioxidative properties (Qiu et al., 2008). In addition, inoculation with *R. etli* greatly enhanced *Phenylalanine AMMONIA-LYASE2* (*PAL2*) expression induced by PspNPS3121 infection. *PAL* genes catalyze the first step in the phenylpropanoid pathway, where L-phenylalanine undergoes deamination to produce trans-cinnamate and ammonia. *PAL* is also involved in cellular defense responses and the formation of lignin, phytoalexins, coumarins, and other flavonoids (Zhang et al., 2017). Furthermore, it has been shown that PAL2 functions in callose deposition (Kohler et al., 2002). *ETHYLENE RESPONSE FACTOR 6* (*ERF6*), a defense activator involved in *Arabidopsis* immunity, is also induced upon pathogen attack *via* the activation of mitogen-activated protein kinases (MAPKs/MPKs) (Huang et al., 2016). *ERF6* also plays a key role in oxidative stress signaling and is necessary for the expression of antioxidant genes in response to biotic and abiotic stresses (Sewelam et al., 2013).

Primed plants responded more efficiently to PspNPS3121 attack by a higher level of superoxide anion production, a faster and stronger accumulation of callose, and a faster activation of plant defense gene expression. In addition, treatment with *R. etli* increased the transcript levels of the ABA-synthesis-related gene *PvAAO3*, after pathogen attack. Suggesting the activation of an ABA-dependent defense mechanism in primed plants (Ton and Mauch-Mani, 2004). Actually, it has been shown that ABA functions as a positive regulator of disease resistance through—for example, potentiation of callose deposition (Ton and Mauch-Mani, 2004; Ton et al., 2005; Flors et al., 2008). Furthermore, environmental conditions such as light, drought, and salt stress have been suggested to regulate ABA biosynthesis (Xiong and Zhu, 2003). This could explain the higher expression levels of the *PvAAO3* gene in *R. etli*–treated plants that were inoculated with water. Thus, ABA displays an important function in plant defense and its task depends on distinctive plant-microorganism interactions (Bari and Jones, 2009).

Therefore, *R. etli* facilitated greater and more rapid activation of defense-related genes following infection with the pathogen, which suggests that priming is an important cellular mechanism in ISR in common bean plants. In addition to the critical role of rhizobacteria in plant growth and development, and in maintaining soil fertility, the indirect biotic stress tolerance effect induced by *R. etli* inoculation should be considered as a major factor influencing the activities of phytopathogenic microorganisms.

Plants exposed to the pathogen in the F1 generation exhibited reduced lesion sizes, low numbers of pathogenic bacterial populations (CFU), and higher transcript levels of *PvERF6* and *PvPAL2*, when compared with the control plants. The results indicate that *R. etli*–primed plants could develop a transgenerational defense memory and could produce offspring that are more resistant to the halo blight disease. However, the accumulations of superoxide anion and callose deposition, in the F1 generation, were much lower than those detected in the F0, and the expression of the *PvAAO3* gene remained without change, after pathogen attack. Notably, up-regulation of *PvERF6* (an ET-independent TF that activates expression of PR genes; Huang et al., 2016) suggests that the transgenerational defense mechanisms implicated in common bean protection mediated by *R. etli* could encompass ET-independent signaling pathway.

## Conclusions

The use of rhizobacteria can stimulate ISR in plants, with the potential to transform modern agriculture; however, research on the exploitation of PGPR remains limited. Considering the positive effects of *R. etli* on crop productivity in the form of biotic stress tolerance and generational and transgenerational inheritances of priming effects, in addition to N-fixation and reduced pesticide application rates, adoption of ISR and PGPR in agriculture should be encouraged as a tool for managing plant stress. Nevertheless, more studies are required to explore the role of root nodule symbiosis and plant innate immunity under stressful conditions such as disease infestation.

## Data Availability Statement

All datasets for this study are included in the manuscript/**Supplementary Files**.

## Author Contributions

RA-V provided the idea of the work. RA-V and AD-V designed the experiments. AD-V and AL-C conducted the experiments and performed the statistical analysis. RA and AD-V participated in the interpretation of results and critically reviewed the manuscript. RA-V wrote the paper. All authors read and approved the final manuscript.

## Funding

This work was supported by Consejo Nacional de Ciencia y Tecnología, grant CB-2015/257129 to RA-V.

## Conflict of Interest

The authors declare that the research was conducted in the absence of any commercial or financial relationships that could be construed as a potential conflict of interest.

## Supplementary Material

Supplementary Figure S1 Transcript levels of genes involved in plant defense as determined by q-PCR at various days after germination (dag). (A) PvWRKY33; (B) PvPAL2; (C) PvERF6. Plants were primed with R. etli, followed by inoculation with P. syringae pv. phaseolicola (Re/Ps), inoculated with the symbiont only (Re/- ; and water, Re/H2O), infected only (-/Ps), or neither primed nor inoculated (control, -/-). Data were normalized to the Tubulin (PvTUB) reference gene. Data are mean ± SD from three independent experiments (n = 3). Statistical significance was determined using two-way ANOVA followed by Tukey’s multiple comparison test, p<0.05. Dag: days after germination. Means with the same letter are not significantly different.

Supplementary Figure S2 Transcript levels of genes involved in plant defense. q-PCR analysis was performed at 16, 18, and 22 days after germination (dag). (A) PvPR1; (B) PvPR10; (C) PvWRKY29, (D) PvWRKY70; (E) PvNPR1; (F) PvMYC2. Plants were primed with R. etli, followed by inoculation with PsNPS3121 (Re/Ps), inoculated only with the symbiont (Re/- ; and water, Re/ H2O), infected only (-/Ps), or neither primed nor inoculated (control, -/-). Data were normalized to the PvActin11 reference gene. Data are mean ± SD from three independent experiments (n = 3). Statistical significance was determined using two-way ANOVA followed by Tukey’s multiple comparison test, p<0.05. Dag: days after germination. Means with the same letter are not significantly different.

Supplementary Figure S3 Transcript levels of genes involved in plant defense. q-PCR analysis was performed at 16, 18, and 22 days after germination (dag). (A) PvAAO3; (B) PvAMI1; (C) PvNIT1; (D) PvBROX2.2. F0 plants were self-pollinated and grown to seed set to generate the F1 progeny. Seeds from the F1 progeny were germinated and exposed to the pathogen only, without rhizobacteria treatment. Samples were obtained at 16, 18, and 22 dag. Data were normalized to the Tubulin (PvTUB) reference gene. Data are mean ± SD from three independent experiments (n = 3). Statistical significance was determined using two-way ANOVA followed by Tukey’s multiple comparison test, p<0.05. Dag: days after germination. Means with the same letter are not significantly different.

Supplementary Figure S4 Transgenerational transcript levels of genes involved in plant defense. (A) PvWRKY33; (B) PvPAL2; (C) PvERF6. F0 plants were self-pollinated and grown to seed set to generate the F1 progeny. Seeds from the F1 progeny were germinated and exposed to the pathogen only, without rhizobacteria treatment. Samples were obtained at 16, 18, and 22 dag. Data were normalized to the Tubulin (PvTUB) reference gene. Data are mean ± SD from three independent experiments (n = 3). Statistical significance was determined using two-way ANOVA followed by Tukey’s multiple comparison test, p<0.05. Dag: days after germination. Means with the same letter are not significantly different.
