# Supplementary figures and images for: Enhancement of Pathogen Resistance in Common Bean Plants by Inoculation With Rhizobium etli
Source: Front Plant Sci. 2019 Oct 22;10:1317. doi: 10.3389/fpls.2019.01317 (PMC6818378; doi:10.3389/fpls.2019.01317)

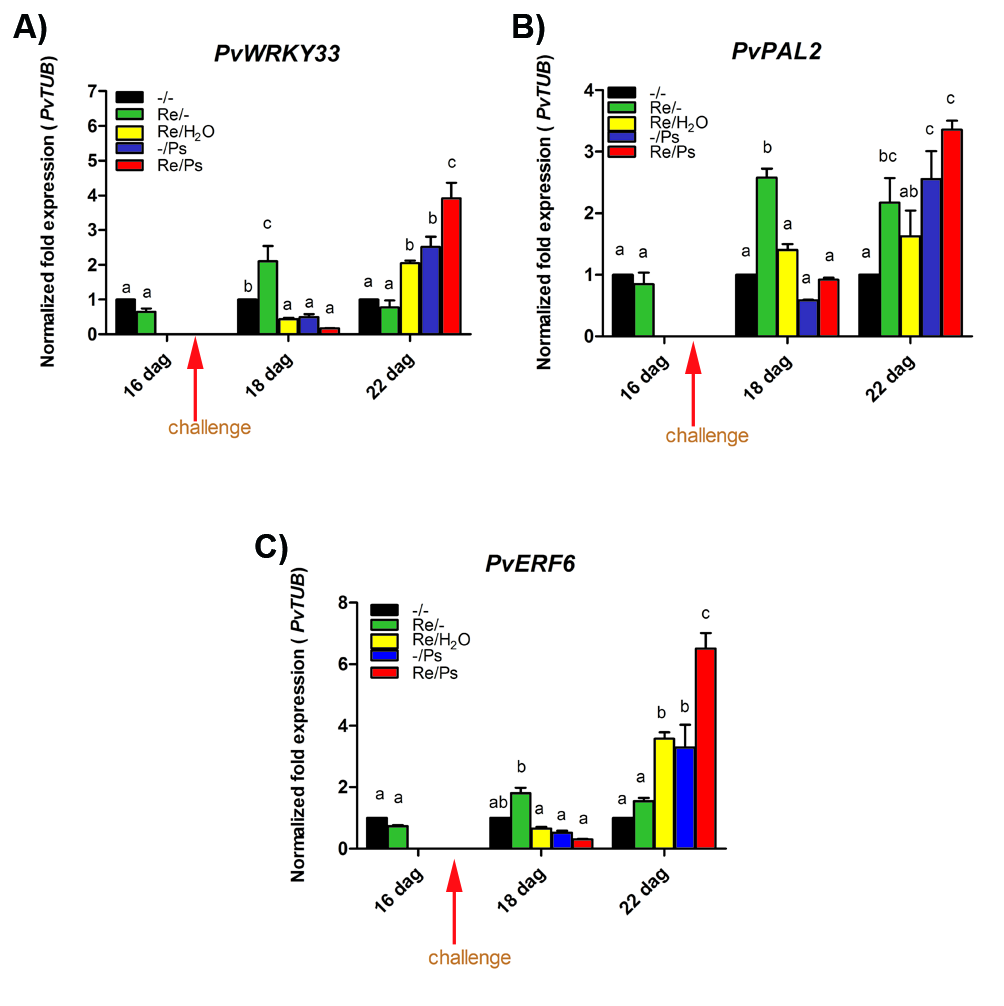

Supplement: Supplementary Figure S1 — Transcript levels of genes involved in plant defense as determined by q-PCR at various days after germination (dag). (A) PvWRKY33; (B) PvPAL2; (C) PvERF6. Plants were primed with R. etli, followed by inoculation with P. syringae pv. phaseolicola (Re/Ps), inoculated with the symbiont only (Re/- ; and water, Re/H2O), infected only (-/Ps), or neither primed nor inoculated (control, -/-). Data were normalized to the Tubulin (PvTUB) reference gene. Data are mean ± SD from three independent experiments (n = 3). Statistical significance was determined using two-way ANOVA followed by Tukey’s multiple comparison test, p<0.05. Dag: days after germination. Means with the same letter are not significantly different. [file Image_1.tif]

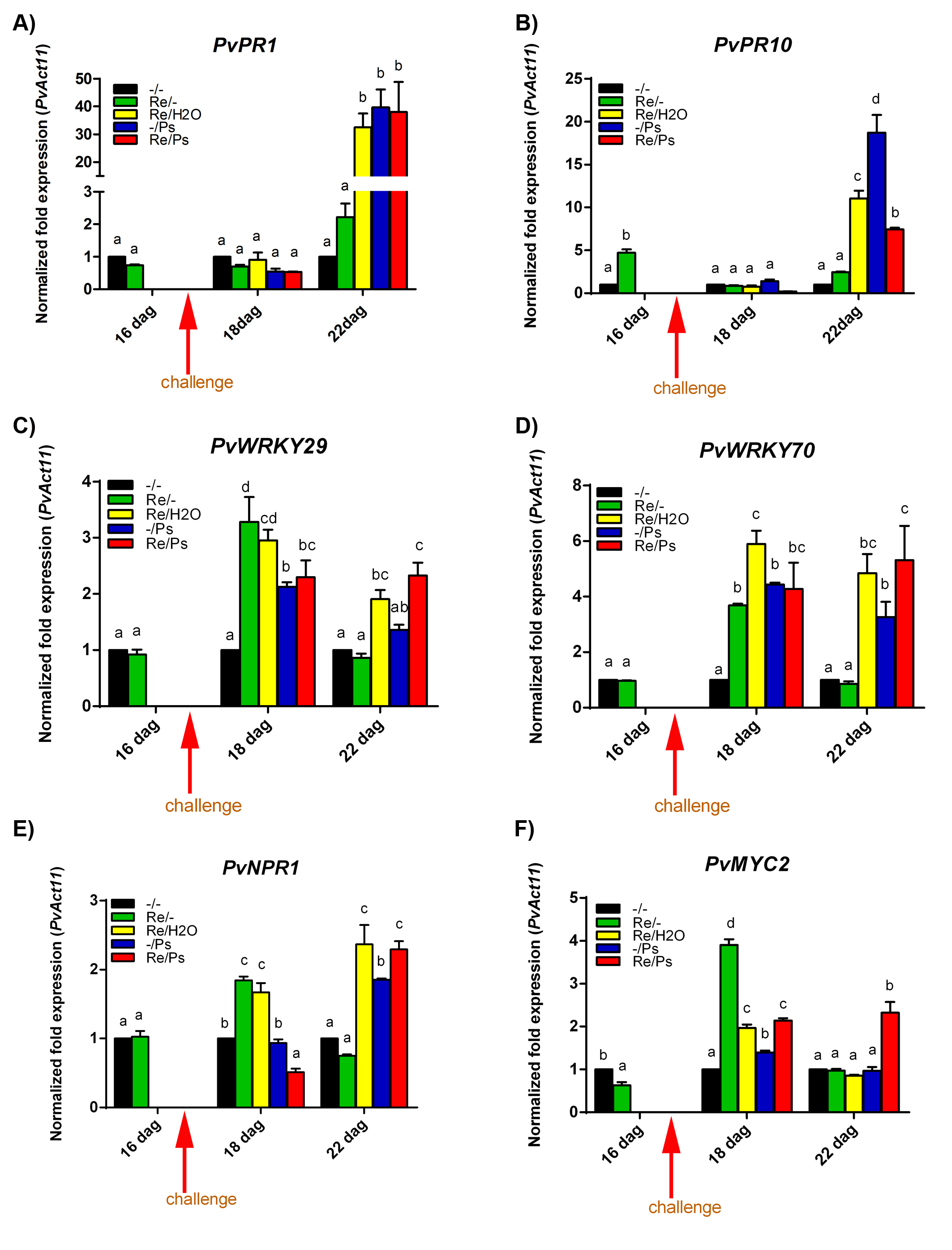

Supplement: Supplementary Figure S2 — Transcript levels of genes involved in plant defense. q-PCR analysis was performed at 16, 18, and 22 days after germination (dag). (A) PvPR1; (B) PvPR10; (C) PvWRKY29, (D) PvWRKY70; (E) PvNPR1; (F) PvMYC2. Plants were primed with R. etli, followed by inoculation with PsNPS3121 (Re/Ps), inoculated only with the symbiont (Re/- ; and water, Re/ H2O), infected only (-/Ps), or neither primed nor inoculated (control, -/-). Data were normalized to the PvActin11 reference gene. Data are mean ± SD from three independent experiments (n = 3). Statistical significance was determined using two-way ANOVA followed by Tukey’s multiple comparison test, p<0.05. Dag: days after germination. Means with the same letter are not significantly different. [file Image_2.tif]

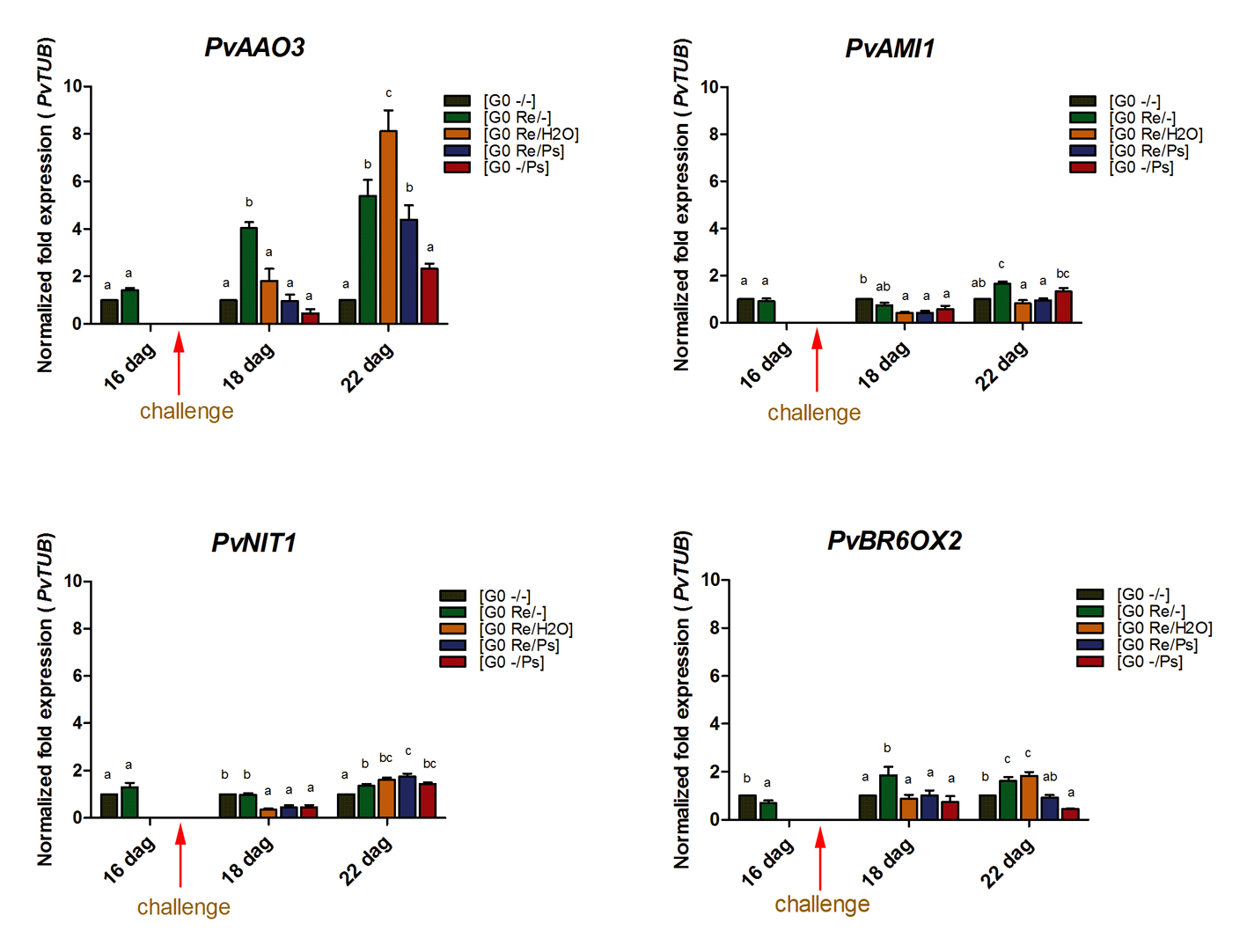

Supplement: Supplementary Figure S3 — Transcript levels of genes involved in plant defense. q-PCR analysis was performed at 16, 18, and 22 days after germination (dag). (A) PvAAO3; (B) PvAMI1; (C) PvNIT1; (D) PvBROX2.2. F0 plants were self-pollinated and grown to seed set to generate the F1 progeny. Seeds from the F1 progeny were germinated and exposed to the pathogen only, without rhizobacteria treatment. Samples were obtained at 16, 18, and 22 dag. Data were normalized to the Tubulin (PvTUB) reference gene. Data are mean ± SD from three independent experiments (n = 3). Statistical significance was determined using two-way ANOVA followed by Tukey’s multiple comparison test, p<0.05. Dag: days after germination. Means with the same letter are not significantly different. [file Image_3.tif]

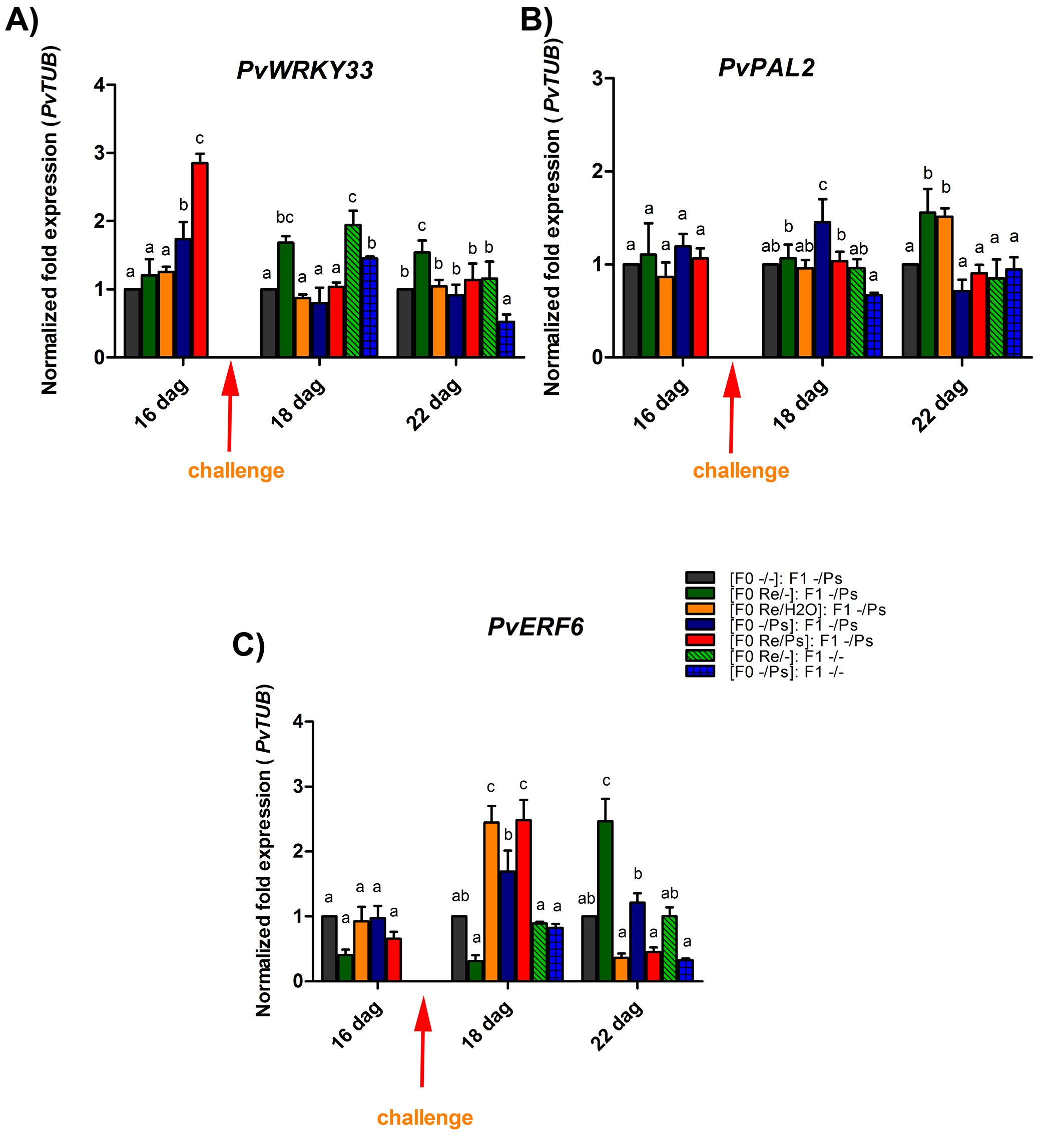

Supplement: Supplementary Figure S4 — Transgenerational transcript levels of genes involved in plant defense. (A) PvWRKY33; (B) PvPAL2; (C) PvERF6. F0 plants were self-pollinated and grown to seed set to generate the F1 progeny. Seeds from the F1 progeny were germinated and exposed to the pathogen only, without rhizobacteria treatment. Samples were obtained at 16, 18, and 22 dag. Data were normalized to the Tubulin (PvTUB) reference gene. Data are mean ± SD from three independent experiments (n = 3). Statistical significance was determined using two-way ANOVA followed by Tukey’s multiple comparison test, p<0.05. Dag: days after germination. Means with the same letter are not significantly different. [file Image_4.tif]
